# How thyme thrives under drought: insights into photosynthetic and membrane-protective mechanisms

**DOI:** 10.1186/s12896-025-01026-9

**Published:** 2025-09-02

**Authors:** Afsoun Kamyab, Davood Samsampour

**Affiliations:** Horticulture Sciences Department, Faculty of Agriculture and Natural Resource, University of Hormozgan, Bandar Abbas, Iran

**Keywords:** Bacterial endophytes, Fungal endophytes, Photosynthesis, Cellular membranes

## Abstract

**Background:**

Drought is an abiotic stress that significantly reduces the yield of thyme (*Thymus vulgaris*). This study investigated how iron oxide nanoparticles (FeNPs), together with symbiotic bacterial (*Azospirillum lipoferum*) and fungal (*Aspergillus oryzae*) endophytes, modulate osmotic adjustment, molecular and biochemical mechanisms related to photosynthesis, and drought tolerance mechanisms in thyme.

**Results:**

The experiment was evaluated as a factorial experiment in a completely randomized design with three replications. evaluating three treatment factors: four irrigation levels (100%, 75%, 50%, and 25% of field capacity), four FeNPs concentrations (0, 0.5, 1, and 1.5 mg L⁻¹), and three endophyte treatments (control, bacterial (EB), and fungal (EF) inoculation). At 25% FC, EB and spraying with 1 mg L^− 1^ FeNPs increased Fv/Fm (maximum quantum efficiency of photosystem II), chlorophyll a, chlorophyll b, and total chlorophyll, carotenoids, relative water content (RWC), and protein levels level protein levels by 18.75%, 10.41%, 31.54%, 18.20%, 14.26%, 35.53%, and 125.22% respectively, compared to the control. At 25% FC, electrolyte leakage (EL) was increased by 47.44% with the combination of EF and 1.5 mg L^− 1^ FeNPs. The highest proline accumulation at 25% FC was observed after inoculation with EF and 1 mg L^− 1^ FeNPs, resulting in significant increases of 36.36% and 13.04%, respectively, compared to the control. Soluble sugar was remarkably increased by 28.57% under upon treatment with FeNPs (1.5 mg L^− 1^ FeNPs). At 25% FC, EB and 1.5 mg L^− 1^ FeNPs showed significant reductions of 17.33% and 37.10%, respectively, in malondialdehyde levels compared to control plants. At 50% FC, 1 mg L⁻¹ FeNPs increased Catalase by 15%, peroxidase by 31.25%, and superoxide dismutase by 43.42%, while higher concentrations reduced enzyme activities. Similarly, 1.5 mg L⁻¹ FeNPs and EB inoculation enhanced ascorbate peroxidase by 37.44% and 17.37%, respectively. FeNPs acted as abiotic stressors at low levels but became toxic at higher concentrations.

**Conclusion:**

Our findings demonstrate that the synergistic application of FeNPs and endophytes significantly enhances drought tolerance in *T. vulgaris* by optimizing photosynthetic efficiency (Fv/Fm, chlorophyll content) and preserving membrane integrity (RWC, MDA reduction). These results provide a framework for leveraging nano-bio partnerships to improve crop resilience under water scarcity.

**Supplementary Information:**

The online version contains supplementary material available at 10.1186/s12896-025-01026-9.

## Background

Drought stress (DS) is a frequent and intense climatic problem affecting farming areas globally, leading to substantial changes in the dispersion of plant flora and considerable decreases in crop harvest outputs. The seasonal annual incidence, strength, and length of drought stress differ in response to altering weather patterns. To address such conditions, plants utilize various physiological, biochemical and structural changes, including stomatal shutdown, alterations in root development and structure, changes in metabolic routes, and modified physiological reactions [1]. Moreover, metabolic alterations and physical and chemical adjustments under drought stress often reduce the lifespan of plants and diminish productivity. However, the effect of drought stress on plants is influenced by the moisture gradient of soil, length and severity of rainfall, type of plants, and growth phase [2, 3]. Drought stress influences.

the development and efficacy of plants, as it lowers moisture levels in plants and can interfere with crucial biological functions such as light synthesis, breathing, and hormonal equilibrium. Drought stress leads to lowered cell pressure, stomatal shutdown, and restricted gas transfer, ultimately reducing development and agricultural yields [4].

Sustainable agriculture utilizing nanobiotechnology possesses significant potential. Nanoparticles (NPs) have become extraordinary resources for enhancing agricultural output in response to intense environmental variations and the increasing severity of drought.

Drought stress adversely affects plant growth and development as well as physiological and metabolic pathways, resulting in disturbances in cellular membranes, antioxidant functions, photosynthetic mechanisms, and nutrient absorption [5]. NPs protect membranes and photosynthetic systems, improve photosynthetic effectiveness, maximize plant hormones and phenolic concentrations, increase nutrient intake and antioxidant activities, and modulate gene activity, thereby reinforcing plants’ tolerance to drought stress [6]. Iron is a crucial component for plants, and its deficiency is prevalent, especially in dry areas. Nanotechnology has been found to have beneficial impacts on plants, and nano products, i.e., NPs and nano-fertilizers, can help enhance physiological and biochemical alterations in plants, thereby boosting their development and antioxidant system function [7]. Iron NPs, particularly under drought-stress circumstances, can optimize iron uptake and enhance the plant’s tolerance to drought stress, aiding the overall development and progress of plants [8, 9].

Many investigations have indicated that FeNPs improve the nutritional condition of plants, increase their tolerance to drought conditions, and enhance their overall development and progress. These NPs function as a supply of iron and by raising nutrient concentrations, they aid in maintaining essential physiological processes such as germination, root activity, and photosynthesis, thereby enhancing the plant’s ability to manage drought stress [10]. Additionally, iron NPs play a key role in strengthening the antioxidant defense mechanism of plants, diminishing the oxidative injury, and maintaining cellular balance. These effects contribute to improving plant development and progress. However, the effect of NPs can differ from growth enhancement to toxicity, depending on their kind, amount, and particular plant varieties involved [11].

In an investigation, the influences of different concentrations of FeNPs and ZnONPs on the morphological characteristics and photosynthetic activity rates of conventional wheat (*Triticum aestivum* L.) were examined. The findings indicated that due to their small dimension, NPs could concentrate in plants and significantly affect their development. Specifically, FeNPs notably increased leaf size while diminishing light capture at specific wavelengths. Conversely, ZnONPs increased chlorophyll b (chl b) concentration.

Nanotechnology has developed nanofertilizers as an effective option to traditional farming practices [12]. Nanofertilizers possess outstanding advantages by promoting major parameters of plant growth, seed germination, growth of shoots and roots, chlorophyll level, stress resistance, and hormonal balance. A key point is that their effectiveness is highly dependent on particle size and application concentration [13]. A study on wheat demonstrated that drought resistance, photosynthesis performance, and grain yield are improved by silica nanoparticles (Nano-SiO_2_). The research indicates that nanoparticles can assist plants in countering environmental stresses such as drought, eventually leading to improved growth in agriculture [14]. Endophytes as tissue colonizers of plants in symbiotic relationships are regulators of their response to external factors and evolutionary associates. Recent evidence indicates their potential to modulate host plant metabolism over and above conventional advantages such as tolerance to stress [15].

Studies have demonstrated that certain endophytes can increase auxin concentrations in stressed plants, thereby promoting their growth. These endophytes assist plants in combating the harmful effects of environmental stresses by modulating the production of reactive oxygen species, regulating metabolic pathways, and strengthening antioxidant defenses [16].

Furthermore, the presence of endophytes in different plant tissues, from roots to leaves and flowers, indicates their ability to establish effective symbiotic relationships with plants and support their survival under severe environmental conditions. Endophytic bacteria (EB) and fungi (EF) are particularly beneficial for plant growth during drought stress, as they stimulate the growth of host plants and play vital roles during periods of drought stress, protecting plants against abiotic factors such as drought, salinity, and high metal toxicity [17, 18]. They achieve this by synthesizing growth regulators and plant hormones that improve plant growth and performance under harsh environmental conditions [19]. Consequently, EB and EF play key roles in influencing plant growth under stressful environments. Therefore, application of endophytes as bio-stimulants in sustainable agriculture, particularly in regions under water stress, can serve as an effective strategy to improve crop production and mitigate the negative effects of climate change on global food security [20].

*Thymus vulgaris*, a Mediterranean aromatic plant, has long been used due to its various medicinal properties. In traditional medicine, this plant has been applied for its antiseptic, antimicrobial, antifungal, antioxidant, and antiviral properties. The importance of thyme as a medicinal plant in addressing various diseases, especially in the field of herbal and natural treatments, is widely acknowledged. The antibacterial and antiviral properties of thyme, particularly in fighting various infections, have made it one of the leading options in medical and agricultural research [21].

Various studies have shown that application of endophytic microorganisms, such as mycorrhizal fungi and bacteria, is highly effective in enhancing plant growth under environmental stresses, including drought and salinity. Specifically, a study examining the impacts of mycorrhizal fungi on maize plants revealed that these fungi, by influencing polyamine metabolism and regulating primary and secondary metabolism, can enhance plants’ tolerance to salt stress [22]. Drought stress induces various physiological and biochemical changes in plants, including the production of reactive oxygen species (ROS), alterations in secondary metabolites, and reduction of photosynthetic efficiency. Numerous studies have explored the mechanisms of plant tolerance to hypoxia and oxygen-limited conditions; and their findings can indirectly contribute to understanding plant responses to drought stress. Specifically, mechanisms such as regulating transcription factors and signaling pathways under hypoxic conditions share similarities with plant responses to drought [23]. In a study, NPs, such as AsA and SeNPs, helped improve plant tolerance to environmental stresses. The results showed that Cr negatively affected plant biomass, gas exchange parameters, total soluble sugars, proline, relative water content, and the expression of antioxidant-related genes, increasing the production of ROS such as MDA, H_2_O_2_, and O_2_^−^, ultimately resulting in reduced plant growth. Similar to the use of SeNPs, which reduced Cr toxicity and improved plant growth under heavy metal stress, FeNPs and endophytes also played significant roles in mitigating the negative effects of drought stress on thyme plants in our study [24]. A study showed that the use of sodium nitroprusside (SNP) as a nitric oxide (NO) donor in plants enhanced their tolerance to various abiotic stresses, including those caused by low temperature and Cr. In our study, FeNPs and endophytes were able to produce similar results in improving thyme performance under drought stress. These results indicated the effective roles of various compounds in regulating the physiological and biochemical responses of plants to environmental stresses [25].

Despite the increasing recognition of the beneficial impacts of (NPs) and endophytes (EB and EF) in enhancing plant resilience under environmental stresses, especially drought, the combined effects of iron oxide NPs and endophytes on improving drought tolerance in *T. vulgaris* have not been fully explored. Previous studies on *T. vulgaris* have mainly focused on its phenolic compounds, flavonoids, and essential oils under drought stress, while physiological traits and antioxidant activities have not been explored [26]. While numerous studies have investigated the individual roles of NPs and endophytes in stress tolerance, there is a lack of research focusing on their synergistic effects in alleviating drought stress, particularly in aromatic and medicinal plants such as thyme. Additionally, the impact of iron oxide NPs on antioxidant responses and physiological processes under drought stress, especially in relation to improving agricultural productivity in water-limited regions, has not been sufficiently studied. Therefore, this research aimed to fill this gap by investigating the combined effects of iron oxide NPs and endophytes on the physiological characteristics, cell membrane integrity, and antioxidant responses of *T. vulgaris* under different irrigation regimes.

Drought stress significantly pose various challenges to global agriculture, including, reducing crop yields and harming plant health. The increasing frequency and severity of droughts, driven by climate change, exacerbate this issue, making it essential to develop effective strategies for enhancing plant drought tolerance. Existing methods, such as conventional irrigation techniques and traditional drought-resistant crop varieties, have shown limited effectiveness in mitigating the damage caused by prolonged water scarcity. Our research aimed to address this problem by investigating the combined effects of iron oxide NPs, EB and EF on improving the drought tolerance of *T. vulgaris*. By focusing on enhancing physiological processes, and antioxidant responses in thyme, this study seeked to offer a promising approach for sustainable agriculture. The application of nanobiotechnology and symbiotic microorganisms could provide a practical and eco-friendly solution to improve crop resilience in water-limited regions, ultimately contributing to higher agricultural productivity and food security.

## Results

### Photosynthetic efficiency and pigments

In this research, drought stress was found to damage the photosynthetic efficiency of plants. The plants grown under irrigation at 50% and 25% FC exhibited the lowest Fv/Fm ratios. The highest Fv/Fm ratio was obtained under 100% FC using 1 mg L^− 1^ Fe NPs along with EB inoculation. Under severe stress conditions (25% FC), the application of 1 mg L^− 1^ FeNPs along with EB and EF inoculation increased Fv/Fm ratio by 18.92% and 18.75%, respectively, compared to the control (Table 1).

**Table 1:** Effects of irrigation levels, bacterial and fungal endophytes and Fe2O3 NPs on chlorophyll and carotenoids content and maximum quantum yield of PSII (Fv/Fm) in *T. vulgaris* L.

Endophyte	Fe_2_O_3_ NPs (mg L^− 1^)	Irrigation (%FC)	Fv/Fm	Chlorophyll a (mg g^− 1^ DW)	Chlorophyll b (mg g^− 1^ DW)	Total Chl (mg g^− 1^ DW)	Carotenoids (mg g^− 1^ DW)
0	0	100	^q−t^ 0.661 ± 0.061	^k−o^ 2.47 ± 0.23	^rs^ 1.55 ± 0.14	^r−v^ 4.02 ± 0.37	^d−l^ 7.97 ± 0.16
		75	^t^ 0.650 ± 0.030	^k−o^ 2.45 ± 0.05	^s^ 1.50 ± 0.08	^tuv^ 3.95 ± 0.13	^i−u^ 7.1 ± 0.65
		50	^u^ 0.617 ± 0.004	^no^ 2.42 ± 0.28	^s^ 1.49 ± 0.07	^uv^ 3.91 ± 0.36	^n−u^ 6.8 ± 0.26
		25	^**u**^ **0.608 ± 0.003**	^**o**^ **2.40 ± 0.23**	^**s**^ **1.49 ± 0.07**	^**v**^ **3.90 ± 0.23**	^**j−u**^ **7.08 ± 0.05**
	0.5	100	^n−t^ 0.674 ± 0.005	^k−o^ 2.46 ± 0.03	^m−r^ 1.64 ± 0.02	^m−v^ 4.10 ± 0.05	^c−i^ 8.09 ± 0.7
		75	^p−t^ 0.667 ± 0.015	^k−o^ 2.45 ± 0.03	^o−s^ 1.58 ± 0.03	^p−v^ 4.04 ± 0.06	^h−s^ 7.36 ± 0.28
		50	^q−t^ 0.660 ± 0.012	^l−o^ 2.44 ± 0.04	^p−s^ 1.58 ± 0.04	^q−v^ 4.02 ± 0.05	^q−u^ 6.56 ± 0.4
		25	^st^ 0.653 ± 0.053	^no^ 2.42 ± 0.20	^rs^ 1.53 ± 0.12	^tuv^ 3.95 ± 0.32	^h−s^ 7.26 ± 0.14
	1	100	^i−o^ 0.696 ± 0.010	^f−l^ 2.56 ± 0.05	^i−o^ 1.72 ± 0.04	^i−o^ 4.28 ± 0.08	^b−e^ 8.73 ± 0.2
		75	^j−p^ 0.693 ± 0.016	^f−n^ 2.53 ± 0.04	^k−r^ 1.66 ± 0.05	^j−r^ 4.19 ± 0.06	^f−o^ 7.69 ± 0.49
		50	^k−q^ 0.685 ± 0.004	^i−o^ 2.49 ± 0.06	^n−s^ 1.60 ± 0.10	^m−v^ 4.09 ± 0.10	^o−u^ 6.68 ± 0.75
		25	^n−t^ 0.675 ± 0.009	^j−o^ 2.47 ± 0.02	^n−s^ 1.61 ± 0.10	^n−v^ 4.08 ± 0.09	^stu^ 6.4 ± 0.16
	1.5	100	^h−n^ 0.699 ± 0.005	^g−o^ 2.52 ± 0.04	^k−r^ 1.66 ± 0.01	^j−s^ 4.19 ± 0.04	^c−g^ 8.46 ± 0.46
		75	^k−q^ 0.686 ± 0.009	^k−o^ 2.45 ± 0.02	^m−s^ 1.62 ± 0.06	^o−v^ 4.07 ± 0.06	^f−o^ 7.69 ± 0.49
		50	^k−r^ 0.682 ± 0.003	^k−o^ 2.45 ± 0.03	^n−s^ 1.61 ± 0.15	^p−v^ 4.06 ± 0.14	^h−t^ 7.18 ± 1.13
		25	^o−t^ 0.671 ± 0.020	^mno^ 2.42 ± 0.02	^qrs^ 1.55 ± 0.09	^s−v^ 3.98 ± 0.11	^tu^ 6.22 ± 0.07
Fungi	0	100	^i−o^ 0.696 ± 0.010	^f−n^ 2.53 ± 0.03	^h−m^ 1.75 ± 0.04	^i−n^ 4.29 ± 0.05	^d−l^ 8.01 ± 0.89
		75	^k−r^ 0.684 ± 0.003	^h−o^ 2.51 ± 0.03	^l−r^ 1.64 ± 0.04	^k−t^ 4.15 ± 0.01	^k−u^ 7.04 ± 1.75
		50	^k−r^ 0.683 ± 0.012	^j−o^ 2.47 ± 0.02	^m−s^ 1.63 ± 0.06	^m−v^ 4.10 ± 0.07	^q−u^ 6.53 ± 0.5
		25	^rst^ 0.658 ± 0.023	^l−o^ 2.44 ± 0.04	^n−s^ 1.59 ± 0.09	^p−v^ 4.06 ± 0.13	^d−k^ 8.01 ± 0.04
	0.5	100	^f−k^ 0.708 ± 0.006	^h−o^ 2.51 ± 0.01	^g−j^ 1.81 ± 0.02	^h−l^ 4.32 ± 0.01	^c−h^ 8.16 ± 0.28
		75	^h−l^ 0.704 ± 0.010	^h−o^ 2.50 ± 0.01	^h−n^ 1.73 ± 0.04	^j−p^ 4.24 ± 0.03	^h−t^ 7.2 ± 0.81
		50	^h−o^ 0.697 ± 0.014	^h−o^ 2.50 ± 0.02	^i−p^ 1.71 ± 0.01	^j−r^ 4.21 ± 0.02	^q−u^ 6.58 ± 0.45
		25	^l−r^ 0.679 ± 0.010	^j−o^ 2.48 ± 0.02	^n−s^ 1.60 ± 0.04	^n−v^ 4.08 ± 0.05	^e−n^ 7.78 ± 0.2
	1	100	^ab^ 0.830 ± 0.009	^b^ 3.20 ± 0.03	^a^ 2.52 ± 0.07	^b^ 5.73 ± 0.09	^bcd^ 8.92 ± 0.06
		75	^c^ 0.790 ± 0.001	^c^ 2.87 ± 0.02	^c−f^ 1.96 ± 0.04	^de^ 4.83 ± 0.06	^f−p^ 7.6 ± 0.38
		50	^def^ 0.733 ± 0.010	^e−j^ 2.59 ± 0.01	^e−h^ 1.87 ± 0.13	^ghi^ 4.46 ± 0.12	^l−u^ 6.97 ± 0.3
		25	^**d−j**^ **0.718 ± 0.018**	^f−n^ 2.54 ± 0.03	^h−m^ 1.75 ± 0.08	^i−m^ 4.29 ± 0.05	^r−u^ 6.49 ± 0.18
	1.5	100	^d−g^ 0.732 ± 0.008	^e−h^ 2.62 ± 0.02	^d−g^ 1.89 ± 0.06	^fgh^ 4.52 ± 0.07	^c−g^ 8.46 ± 0.5
		75	^d−i^ 0.720 ± 0.011	^f−l^ 2.55 ± 0.02	^g−j^ 1.81 ± 0.03	^hij^ 4.36 ± 0.04	^h−r^ 7.43 ± 0.4
		50	^j−p^ 0.693 ± 0.002	^f−m^ 2.54 ± 0.03	^j−q^ 1.69 ± 0.01	^j−p^ 4.24 ± 0.02	^m−u^ 6.85 ± 1.17
		25	^m−s^ 0.677 ± 0.001	^j−o^ 2.47 ± 0.02	^m−s^ 1.62 ± 0.03	^m−v^ 4.10 ± 0.05	^m−u^ 6.88 ± 0.14
Bacteria	0	100	^d−j^ 0.718 ± 0.006	^f−l^ 2.55 ± 0.01	^g−k^ 1.79 ± 0.03	^h−k^ 4.34 ± 0.04	^b−f^ 8.57 ± 1.31
		75	^h−l^ 0.705 ± 0.013	^h−o^ 2.51 ± 0.02	^g−l^ 1.78 ± 0.03	^i−m^ 4.29 ± 0.05	^g−q^ 7.54 ± 0.65
		50	^n−t^ 0.674 ± 0.003	^k−o^ 2.46 ± 0.01	^k−r^ 1.66 ± 0.04	^l−u^ 4.12 ± 0.03	^k−u^ 7.04 ± 0.05
		25	^o−t^ 0.671 ± 0.001	^k−o^ 2.45 ± 0.07	^n−s^ 1.61 ± 0.07	^p−v^ 4.06 ± 0.01	^o−t^ 6.7 ± 0.09
	0.5	100	^d−i^ 0.722 ± 0.002	^efg^ 2.64 ± 0.03	^cde^ 2.00 ± 0.07	^efg^ 4.65 ± 0.09	^bc^ 9.06 ± 0.07
		75	^d−i^ 0.720 ± 0.014	^f−l^ 2.56 ± 0.01	^g−k^ 1.79 ± 0.11	^h−k^ 4.35 ± 0.11	^c−i^ 8.09 ± 0.9
		50	^h−m^ 0.703 ± 0.010	^f−k^ 2.57 ± 0.01	^k−r^ 1.66 ± 0.09	^j−r^ 4.23 ± 0.10	^g−q^ 7.51 ± 1.04
		25	^g−l^ 0.706 ± 0.009	^f−n^ 2.53 ± 0.02	^j−p^ 1.70 ± 0.06	^j−q^ 4.23 ± 0.08	^p−u^ 6.60 ± 0.06
	1	100	^**a**^ **0.852 ± 0.007**	^**a**^ **3.42 ± 0.03**	^**a**^ **2.59 ± 0.06**	^**a**^ **6.02 ± 0.08**	^**a**^ **10.8 ± 0.34**
		75	^bc^ 0.814 ± 0.005	^b^ 3.13 ± 0.03	^b^ 2.20 ± 0.06	^c^ 5.34 ± 0.09	^bcd^ 8.96 ± 0.05
		50	^d^ 0.743 ± 0.007	^cd^ 2.81 ± 0.02	^bc^ 2.07 ± 0.07	^d^ 4.89 ± 0.08	^c−i^ 8.09 ± 0.08
		25	^**d−h**^ **0.722 ± 0.010**	^**ef**^ **2.65 ± 0.03**	^**c−f**^ **1.96 ± 0.10**	^**fg**^ **4.61 ± 0.12**	^**u**^ **6.09 ± 0.12**
	1.5	100	^c^ 0.801 ± 0.003	^c^ 2.84 ± 0.01	^cd^ 2.01 ± 0.04	^de^ 4.85 ± 0.05	^ab^ 9.53 ± 0.34
		75	^c^ 0.799 ± 0.009	^de^ 2.69 ± 0.01	^cde^ 1.98 ± 0.27	^def^ 4.68 ± 0.28	^b−e^ 8.74 ± 0.26
		50	^de^ 0.736 ± 0.021	^e−i^ 2.61 ± 0.02	^f−i^ 1.84 ± 0.13	^ghi^ 4.46 ± 0.11	^e−m^ 7.81 ± 1.30
		25	^e−j^ 0.713 ± 0.011	^f−n^ 2.53 ± 0.02	^f−i^ 1.84 ± 0.08	^hij^ 4.37 ± 0.09	^u^ 6.13 ± 0.32

Means followed by the same letter are not significantly different according to the least significant difference (LSD) test at *P* ≤ 0.05

In this investigation, the plants treated with EB and 1 mg L^− 1^ Fe NPs under irrigation at 100% FC showed the highest levels of chl a, chl b, chl t, and carotenoid (Table 1). As expected, reduced irrigation levels decreased chl and carotenoid contents. EB and EF, along with Fe NPs foliar spraying, played positive roles in maintaining chl and carotenoid contents of thyme under drought-stress conditions. Under severe drought stress (25% FC), combined application of EB inoculation and 1 mg L^− 1^ Fe NPs spraying increased chl a, chl b, chl t and carotenoids by 10.41%, 31.54%, 18.20%, and 18.21,% respectively, compared to the control group. Additionally, with the increase of Fe NPs concentration from 1 to 1.5 mg L^− 1^, the level of photosynthetic pigments was decreased (Table 1).

### Physiological characteristics

Electrolyte leakage (El) is used to assess membrane permeability and its increase indicates stress and potential damage to cell membrane. In the present study, increasing the levels of drought stress significantly increased electrolyte leakage in plants. EB and EF, along with Fe NPs, played key roles in modulating electrolyte leakage under stress conditions. Under severe stress conditions, simultaneous use of fungal inoculation and foliar spraying of 1.5 mg L^− 1^ Fe NPs reduced electrolyte leakage by 47.44% compared to the control (Table 2).

**Table 2:** Effects of irrigation levels, bacterial and fungal endophytes and Fe_2_O_3_ NPs on physiological characteristics in *T. vulgaris* L.

Endophyte	Fe_2_O_3_ NPs (mg L^− 1^)	Irrigation (%FC)	Electrolyte leakage (%)	RWC (%)
0	0	100	^qrs^ 10.73 ± 1.09	^ab^ 84.69 ± 3.83
		75	^hij^ 22.81 ± 1.5	^fgh^ 74.72 ± 2.42
		50	^cd^ 32.74 ± 3.52	^ijk^ 62.66 ± 3.21
		25	^ab^ 40.7 ± 1.57	^mn^ 48.66 ± 3.51
	0.5	100	^qrs^ 11.14 ± 1	^a^ 85.46 ± 1.27
		75	^ijk^ 21.7 ± 3.12	^e−h^ 75.42 ± 3.06
		50	^cde^ 32.41 ± 2.12	^k^ 60.42 ± 2.37
		25	^ab^ 39.87 ± 2.01	^mn^ 48.75 ± 3.52
	1	100	^qrs^ 11.05 ± 1.23	^a^ 85.94 ± 2.07
		75	^hij^ 22.8 ± 3.3	^gh^ 74.10 ± 1.94
		50	^cde^ 32.47 ± 1.41	^k^ 60.60 ± 5.5
		25	^ab^ 38.52 ± 3.11	^**mn**^ **49.18 ± 2.72**
	1.5	100	^qrs^ 10.95 ± 1.07	^ab^ 84.52 ± 0.9
		75	^hi^ 23.22 ± 2.46	^h^ 72.46 ± 3.22
		50	^cde^ 32.41 ± 1.42	^ijk^ 62.81 ± 6.41
		25	^**b**^ **38.49 ± 3.74**	^n^ 46.21 ± 2.63
Fungi	0	100	^p−s^ 11.85 ± 3.21	^b−e^ 79.8 ± 4.15
		75	^ijk^ 21.04 ± 1.15	^e−h^ 75.44 ± 3.6
		50	^cd^ 34.18 ± 2.64	^ijk^ 62.04 ± 3
		25	^a^ 41.8 ± 1.5	^lm^ 51.87 ± 3.37
	0.5	100	^qrs^ 10.92 ± 0.27	^a^ 85.04 ± 2.69
		75	^jkl^ 19.63 ± 1.18	^e−h^ 75.52 ± 3.13
		50	^ef^ 29.3 ± 0.6	^ijk^ 65.29 ± 2.5
		25	^c^ 35.09 ± 1.65	^l^ 55.39 ± 3.6
	1	100	^qrs^ 10.79 ± 0.78	^abc^ 83.97 ± 3.32
		75	^lmn^ 17.52 ± 1.3	^d−g^ 78.43 ± 3.18
		50	^gh^ 25.75 ± 1.97	^h^ 72.73 ± 3.26
		25	^de^ 31.74 ± 1.63	^i^ 65.84 ± 1.88
	1.5	100	^rs^ 9.82 ± 0.21	^abc^ 83.51 ± 3.59
		75	^o−r^ 13.07 ± 1.89	^e−h^ 75.77 ± 2.34
		50	^nop^ 15.04 ± 0.93	^fgh^ 74.47 ± 3.5
		25	^**ijk**^ **21.39 ± 1.98**	^ijk^ 65.18 ± 3.54
Bacteria	0	100	^qrs^ 10.48 ± 1.28	^a−d^ 82.29 ± 2.14
		75	^hi^ 23.19 ± 4.41	^gh^ 74.41 ± 3.16
		50	^cd^ 33.4 ± 1.44	^k^ 60.73 ± 1.62
		25	^ab^ 40.40 ± 3.7	^lm^ 51.44 ± 2.21
	0.5	100	^qrs^ 10.58 ± 0.87	^abc^ 84 ± 3.6
		75	^klm^ 19.07 ± 2.77	^d−g^ 78.50 ± 0.5
		50	^fg^ 27.95 ± 2.62	^e−h^ 76.18 ± 2.55
		25	^c^ 35.14 ± 2.01	^ijk^ 62.33 ± 3.05
	1	100	^s^ 9.68 ± 0.71	^**a**^ **86.19 ± 1.59**
		75	^lmn^ 17.12 ± 2.01	^e−h^ 76.82 ± 1.04
		50	^hi^ 23.74 ± 0.65	^e−h^ 75.19 ± 3.01
		25	^cd^ 33.08 ± 1.12	^**ij**^ **65.95 ± 3.42**
	1.5	100	^qrs^ 10.49 ± 0.63	^a^ 85.02 ± 2.6
		75	^opq^ 13.41 ± 0.71	^c−f^ 79.40 ± 1.43
		50	^mno^ 16.19 ± 1.05	^e−h^ 76.78 ± 1.11
		25	^ijk^ 22.23 ± 2.39	^jk^ 61.25 ± 4.23

Means followed by the same letter are not significantly different according to the least significant difference (LSD) test at *P* ≤ 0.05

Under stress conditions, plants show lower relative water content (RWC) compared to normal irrigation. The simultaneous use of Fe NPs, EB, and EF was found to help maintain RWC. The highest RWC was observed under 100% FC and 25% FC with EB treatment and foliar spraying of 1 mg L^− 1^ Fe NPs, reaching 86.19% and 65.95%, respectively. The highest percentage increase of RWC compared to the control was observed under 25% FC with EB treatment and foliar spraying of 1 mg L^− 1^ Fe NPs, with the value of 35.53% (Table 2).

**Fig. 1:**
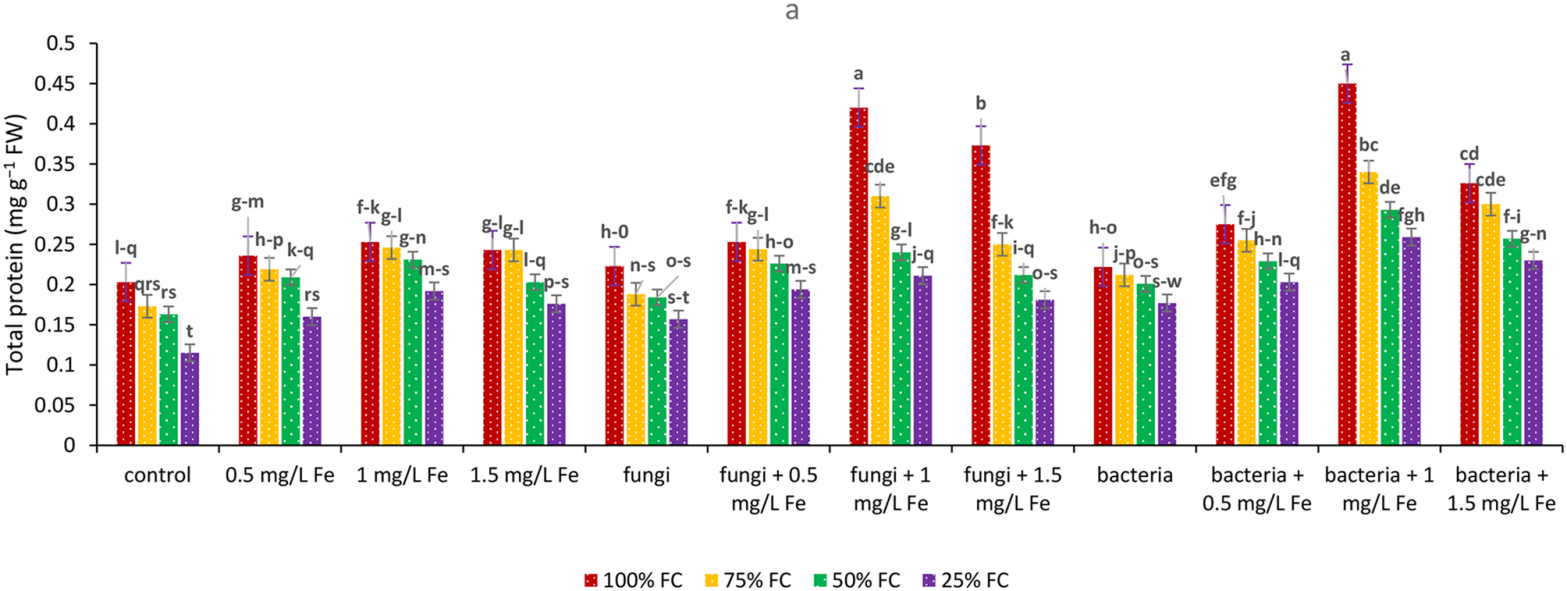
Effects of endophytes (E) and Fe_2_O_3_ NPs (F) and drought (D) on Total protein content of *T. vulgaris* L. (E*F*D).Means followed by the same letter are not significantly different according to the least significant difference (LSD) test at *P* ≤ 0.05

By decreasing irrigation level, protein content was significantly decreased. During severe drought stress, Fe NPs and endophytes played important roles in increasing protein content, such that at 25% FC, EB and 1 mg L^− 1^ Fe NPs, protein content in thyme was increased by 125.22%. The highest protein content was achieved under 100% FC with EB treatment and 1 mg L^− 1^ Fe NPs (Fig. 1).

**Fig. 2:**
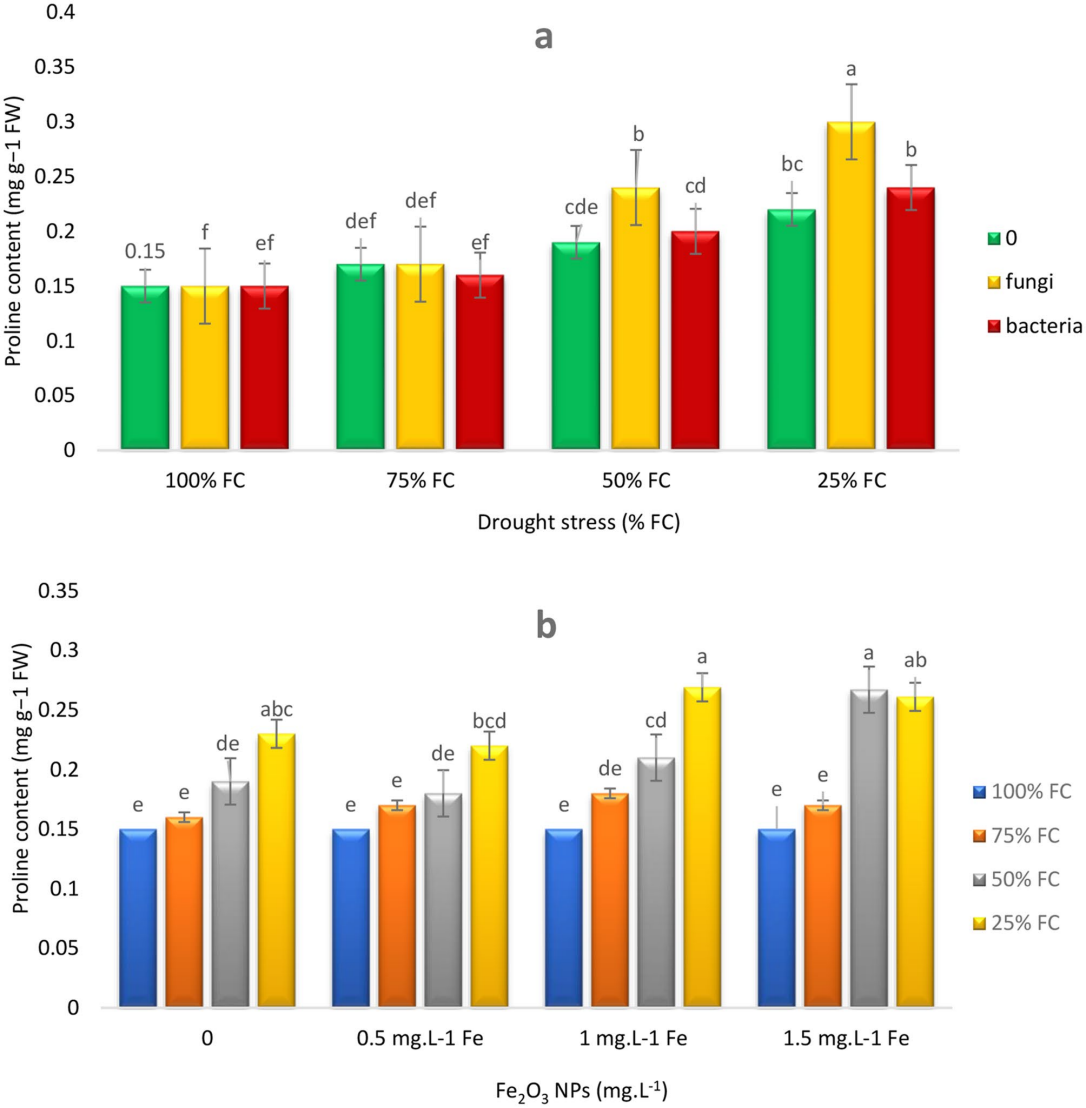
Effects of endophytes and drought (E*D) (**a**), Fe_2_O_3_ NPs and drought (F*D) (**b**), on proline content of *T. vulgaris* L. Means followed by the same letter are not significantly different according to the least significant difference (LSD) test at *P* ≤ 0.05

As expected, drought stress increased proline accumulation in thyme plants. The results showed that endophytes and Fe NPs significantly contributed to further increase of proline accumulation under drought stress. Inoculation of plants with EF significantly increased proline accumulation (36.36%) and inoculation with EB increased it by 9.09% under severe drought stress (25% FC) (Fig. 2a). Additionally, foliar spraying of plants with 1 mg L^− 1^ Fe NPs increased proline accumulation by 13.04% under severe drought stress, while increasing Fe NPs concentration to 1.5 mg L^− 1^ did not change proline amount (Fig. 2b).

**Fig. 3:**
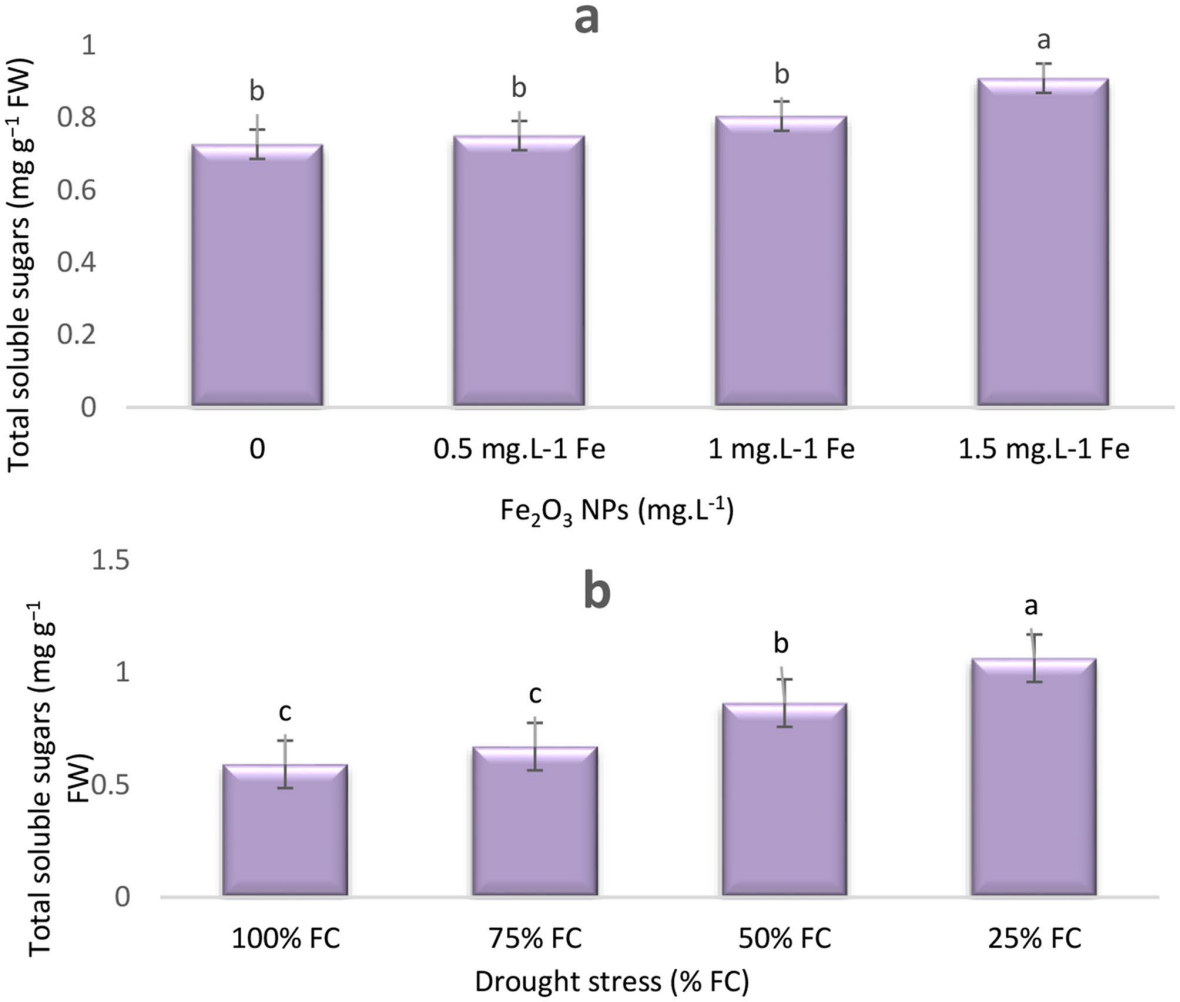
Effects of Fe_2_O_3_ NPs (**a**) and drought stress (**b**) on total soluble sugars of *T. vulgaris* L. Means followed by the same letter are not significantly different according to the least significant difference (LSD) test at *P* ≤ 0.05

Overall, decrease of irrigation levels significantly increased soluble sugar content in plants (Fig. 3). Under moderate (50% FC) and severe (25% FC) stress, endophytes increased the accumulation of soluble sugars in plants. However, this increase was not statistically significant. Treatment with 1.5 mg L^− 1^ Fe NPs and at 25% FC resulted in a significant increase by 28.57% and 2.12 fold, respectively, compared to the control (Figs. 3a, b).

**Fig. 4:**
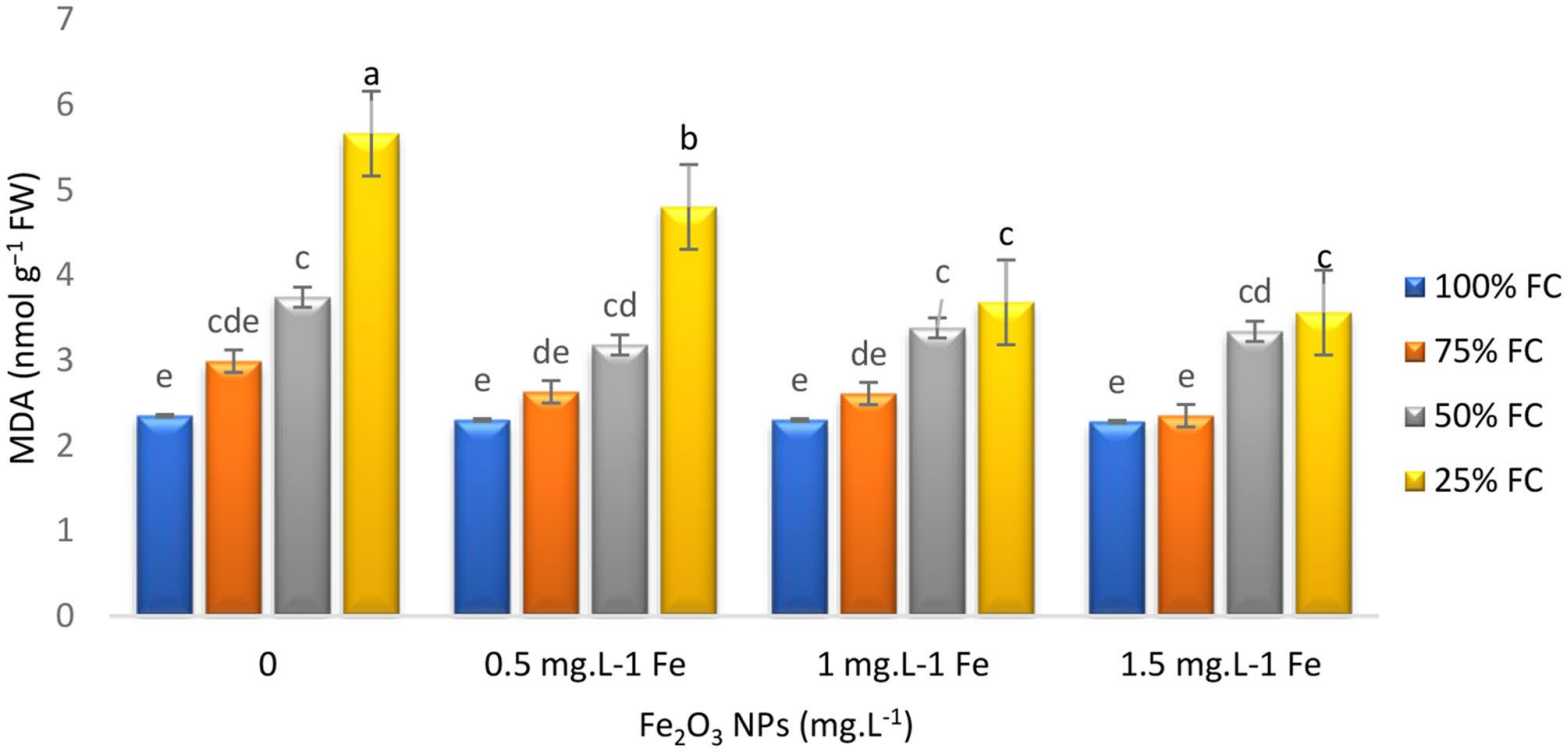
Effects of Fe_2_O_3_ NPs and drought (F*D) on MDA content of *T. vulgaris* L. Means followed by the same letter are not significantly different according to the least significant difference (LSD) test at *P* ≤ 0.05

Measuring MDA levels can be used as an assessment tool to indicate cell damage caused by lipid peroxidation in plants under stress conditions. Research findings have shown that irrigation levels, endophytes, and Fe NPs had significant effects on MDA contents in thyme plants. Under moderate and severe stress conditions, endophytes and Fe NPs decreased MDA contents in plants. Under 25% FC irrigation, EB and EF reduced MDA content by 17.33% and 14.91%, respectively (Fig. 4a). Additionally, plants treated with 1 and 1.5 mg L^− 1^ Fe NPs showed 34.98% and 37.10% lower MDA contents, respectively, compared to control plants under severe stress conditions (Fig. 4b). These findings indicated the positive effects of inoculated endophytes and Fe NPs in enhancing the drought stress tolerance of thyme plants.

### Antioxidant properties

**Fig. 5:**
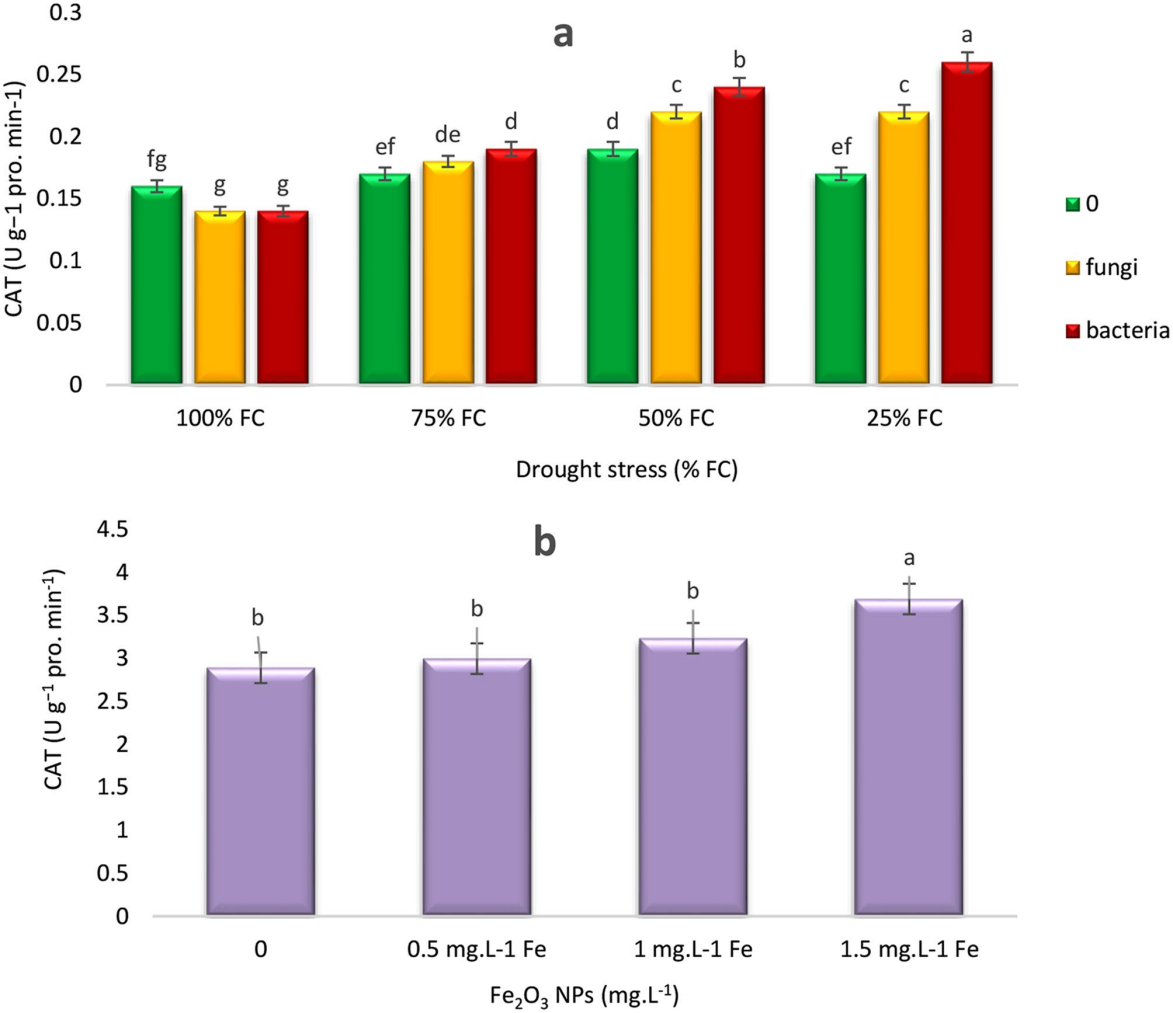
Effects of endophytes and drought (F*D) (**a**) and Fe_2_O_3_ NPs (**b**) on CAT activity of *T. vulgaris* L. under four different irrigation levels. Means followed by the same letter are not significantly different according to the least significant difference (LSD) test at *P* ≤ 0.05

**Fig. 6:**
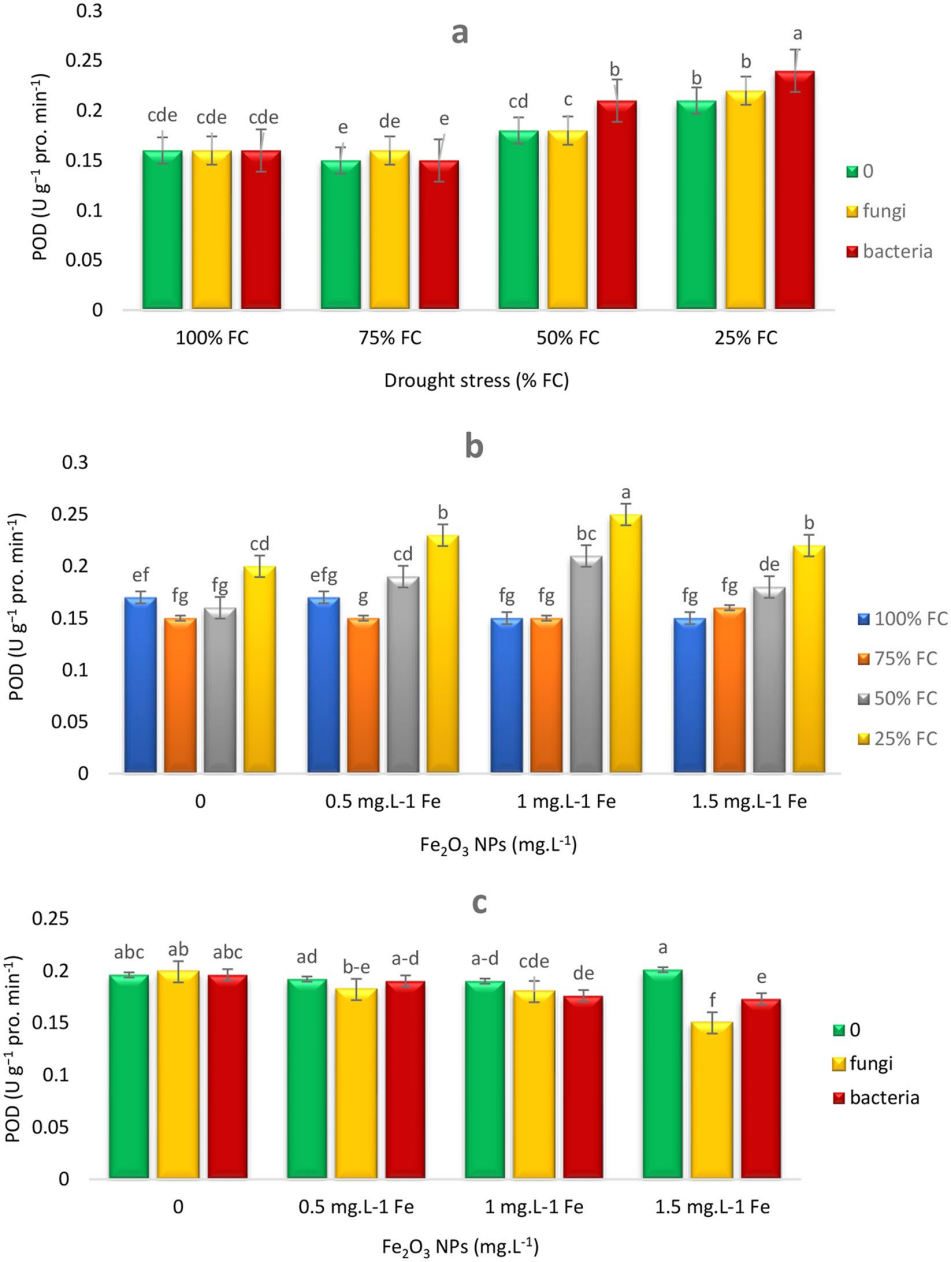
Effects of endophytes and drought (E*D) (**a**), Fe_2_O_3_ NPs and drought (F*D) (**b**), endophytes and Fe_2_O_3_ NPs (E*F) (**c**) on POD activity of *T. vulgaris* L. Means followed by the same letter are not significantly different according to the least significant difference (LSD) test at *P* ≤ 0.05

**Fig. 7:**
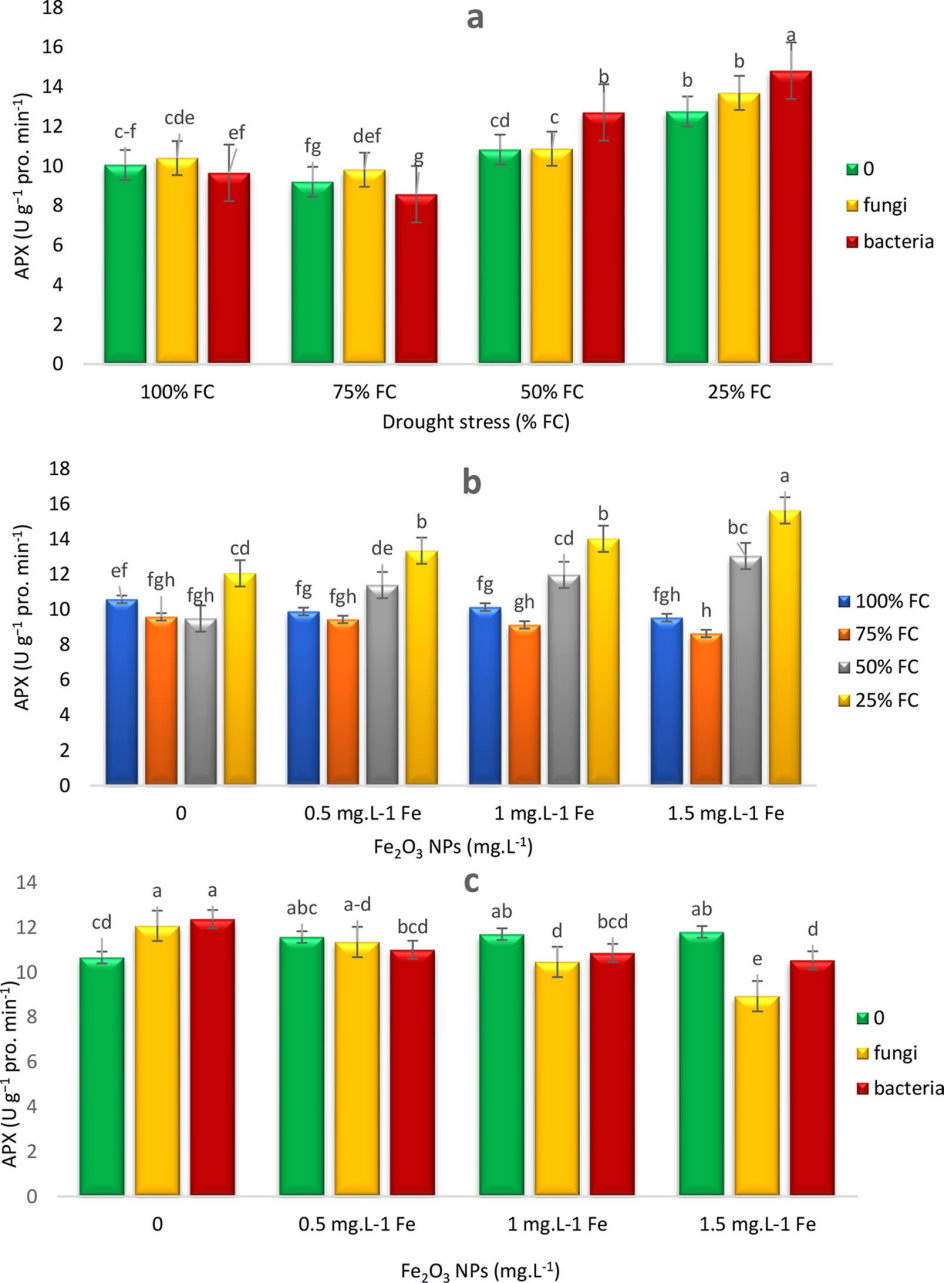
Effects of endophytes and drought (E*D) (**a**), Fe_2_O_3_ NPs and drought (F*D) (**b**), endophytes and Fe_2_O_3_ NPs (E*F) (c) on) on APX activity of *T. vulgaris* L. Means followed by the same letter are not significantly different according to the least significant difference (LSD) test at *P* ≤ 0.05

**Fig. 8:**
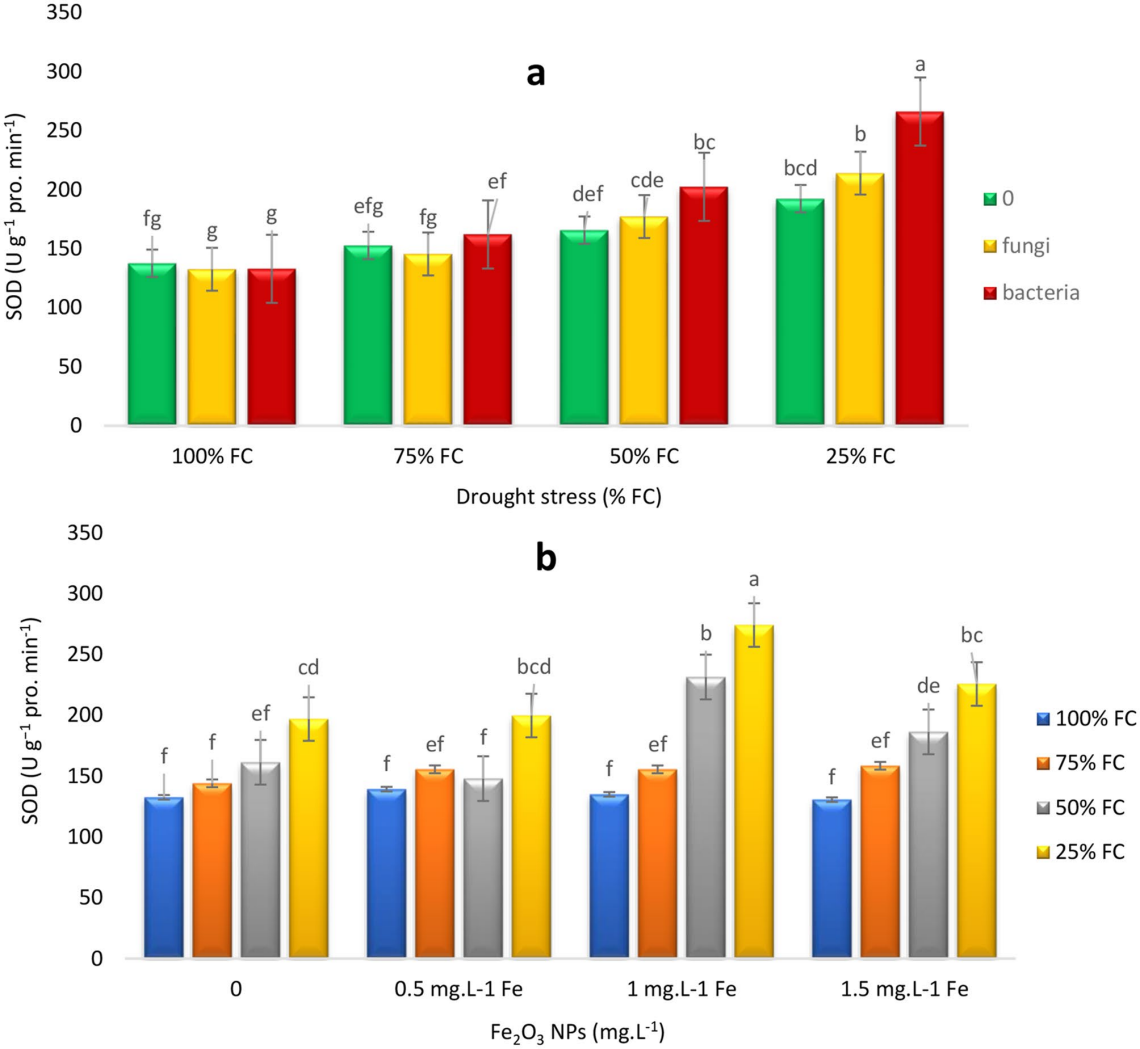
Effects of endophytes and drought (E*D) (**a**), Fe_2_O_3_ NPs and drought (F*D) (**b**), on SOD activity of *T. vulgaris* L. Means followed by the same letter are not significantly different according to the least significant difference (LSD) test at *P* ≤ 0.05

Figures 5, 6, 7 and 8 depict the changes of antioxidant enzyme activities of thyme in response to drought stress, endophytes, and Fe NPs. Catalase (CAT) activity at 100% FC level indicated optimal water conditions, CAT activity at 100% FC was lower compared to other drought levels.This reflected low stress and reduced need for antioxidant CAT activity in plants. With the decrease of moisture level to 75% and 50% FC, CAT activity increased across all three treatments. At 25% FC level, representing the highest drought stress, CAT activity was declined, but the most effective treatment was EB, which significantly increased CAT activity by 36.84%, while CAT levels remained constat under EF treatment but showed better performance than the control (0) (Fig. 5a).

As soil moisture was decreased from 100 to 50% FC, CAT activity was increased, reaching its highest level at 50% FC and then, decreased as moisture levels dropped to 25% FC. It was also revealed that with the increase of Fe NPs concentration to 1 mg L^− 1^ under 50% FC, CAT activity was increased by 15%, though this increase was not significant, and then decreased at 1.5 mg L^− 1^ Fe NPs. The graph (Fig. 5b) indicated that both soil moisture and Fe NPs concentration affected CAT activity, with soil moisture having a more pronounced effect. With the increase of Fe NPs concentration to 1.5, CAT activity significantly increased and reached its highest level.

At 100% FC, the activity of Peroxidase (POD) remained constant across all treatments (including control, EB, and EF). This finding indicated that under optimal irrigation conditions, the presence of endophytes had no significant effect on POD activity. As the field capacity was decreased to 75% FC, POD activity was decreased in control and EB treatments, while in EF treatment, POD activity was maintained constant. This result showed that EF provided greater protective effects on POD activity at this water stress level. At 50% FC, POD activity was increased by 16.67% in EB treatment, while its activity remained constant in EF and control treatments. Finally, at the most severe level of water stress, i.e. 25% FC, POD activity was increased by 14.28% and 4.76% in EB and EF treatments, respectively. This increase indicated that EB had a greater effect than EF in enhancing POD activity under severe water stress conditions (Fig. 6a). At 100% FC, addition of Fe NPs had little effect on POD levels and the amount of POD remained constant. At 75% FC, increasing Fe NPs concentration from 0.5 to 1.5 mg L⁻¹ resulted in slight increases in POD levels. At 50% and 25% FC, increasing Fe NPs concentration to 1.5 mg L⁻¹ caused significant increases in POD levels by 31.25% and 25%, respectively, but further increase of Fe NPs concentration decreased POD levels (Fig. 6b). Under EB and 0.5 mg L^− 1^ Fe NPs conditions, the amount of POD was significantly increased, while higher concentrations of Fe NPs decreased the amount of POD (Fig. 6c).

As moisture was decreased from 100 to 50% and then to 25% FC, ascorbate peroxidase (APX) levels were continuously increased in both EB and EF treatments. Reduction of APX enzyme activity at 75% FC indicated that moderate moisture reduction decreased APX activity. When moisture was decreased to 50% and 25% FC, APX enzyme activity was increased under both EB and EF treatments. EB treatment caused significant increases in APX activity by 17.37% and 16.15% at 50% and 25% FC, respectively (Fig. 7a).

Under 100% FC conditions, APX levels were initially decreased by increasing Fe NPs concentration, then increased at 1 mg L⁻¹, and finally decreased once more when Fe NPs concentration was raised to 1.5 mg L⁻¹. Overall, the results indicated that reducing moisture to 50% and 25% FC increased APX levels. At these stress levels, Fe NPs treatments, especially at higher concentrations (1 and 1.5 mg L⁻¹), resulted in significant increases in APX enzyme activity. In contrast, under 100% and 75% FC conditions, APX levels were either decreased by increasing Fe NPs concentrations or showed only minor changes, indicating that water deficit conditions, combined with the use of Fe NPs, enhanced APX activity. At 50% and 25% FC, 1.5 mg L⁻¹ Fe NPs treatment significantly increased APX levels by 37.44% and 29.62%, respectively (Fig. 7b). Under EF and 0.5 mg L^− 1^ Fe NPs conditions, the amount of APX was significantly increased, while higher concentrations of Fe NPs decreased the amount of APX (Fig. 7c).

At 100% FC, the activity of superoxide dismutase) SOD) enzyme was constant across all three treatments (control, EB, and EF) and no significant differences were observed among them. This indicated that under optimal moisture conditions, there was less oxidative stress in plants and there was no need for strong activation of enzymatic defense systems such as SOD. As the moisture level was decreased to 75% FC, SOD activity was slightly increased in all three treatments. This increase suggested that with relative reduction of moisture, plants began to activate their defense systems to cope with new stress conditions. At 50% FC, SOD activity was significantly increased. In 50% FC, EB treatment resulted in the highest SOD activity (22.15%). This finding indicated that under the conditions of severe moisture reduction, EB treatment had a positive effect in strengthening the defense system of the plants. At 25% FC, SOD activity reached its highest level in all treatments, with EB treatment showing the highest SOD activity, accounting for an increase of 38.37% compared to the control plants (Fig. 8a).

Under 100% FC conditions, SOD enzyme activity did not show significant differences across the four concentrations of Fe NPs (0, 0.5, 1, and 1.5 mg L⁻¹). At 75% FC, SOD activity was slightly increased, but there were no significant differences among different Fe NPs treatments. At 50% and 25% FC, SOD activity was significantly increased by 43.42% and 39.25% at 1 mg L⁻¹ concentration, respectively, and then decreased at 1.5 mg L⁻¹ (Fig. 8b).

### Drought tolerance test

The obtained results indicated that increasing PEG concentrations reduced the growth of isolates in the culture medium, suggesting a decrease in their drought tolerance. Different isolates exhibited different responses to drought stress based on their genetic and biochemical characteristics. Among the 60 bacterial and 72 fungal strains analyzed, those with superior characteristics, including rapid growth and high tolerance to desiccation, were selected for further investigation. These selected strains demonstrated robust growth even under low water potential conditions (-9 bars). Isolates that exhibited superior growth at higher PEG concentrations and did not show significant reductions in growth parameters were identified as drought-tolerant isolates. These isolates were chosen for further research and practical applications due to their ability to withstand drought stress, emphasizing their potential to be used in real agricultural conditions under water scarcity. After selecting these isolates, their genetic sequences were obtained through sanger sequencing and deposited into the GenBank database. The selected strains were assigned accession numbers: *Azospirillum lipoferum* (PP837752) and *Aspergillus oryzae* (PP840055). The findings of this study suggested that the selected isolates could serve as promising candidates for developing tolerant bio-products or establishing symbiotic relationships with plants in water-limited environments.

### Correlation and principal component analysis

#### HCA analysis

**Fig. 9:**
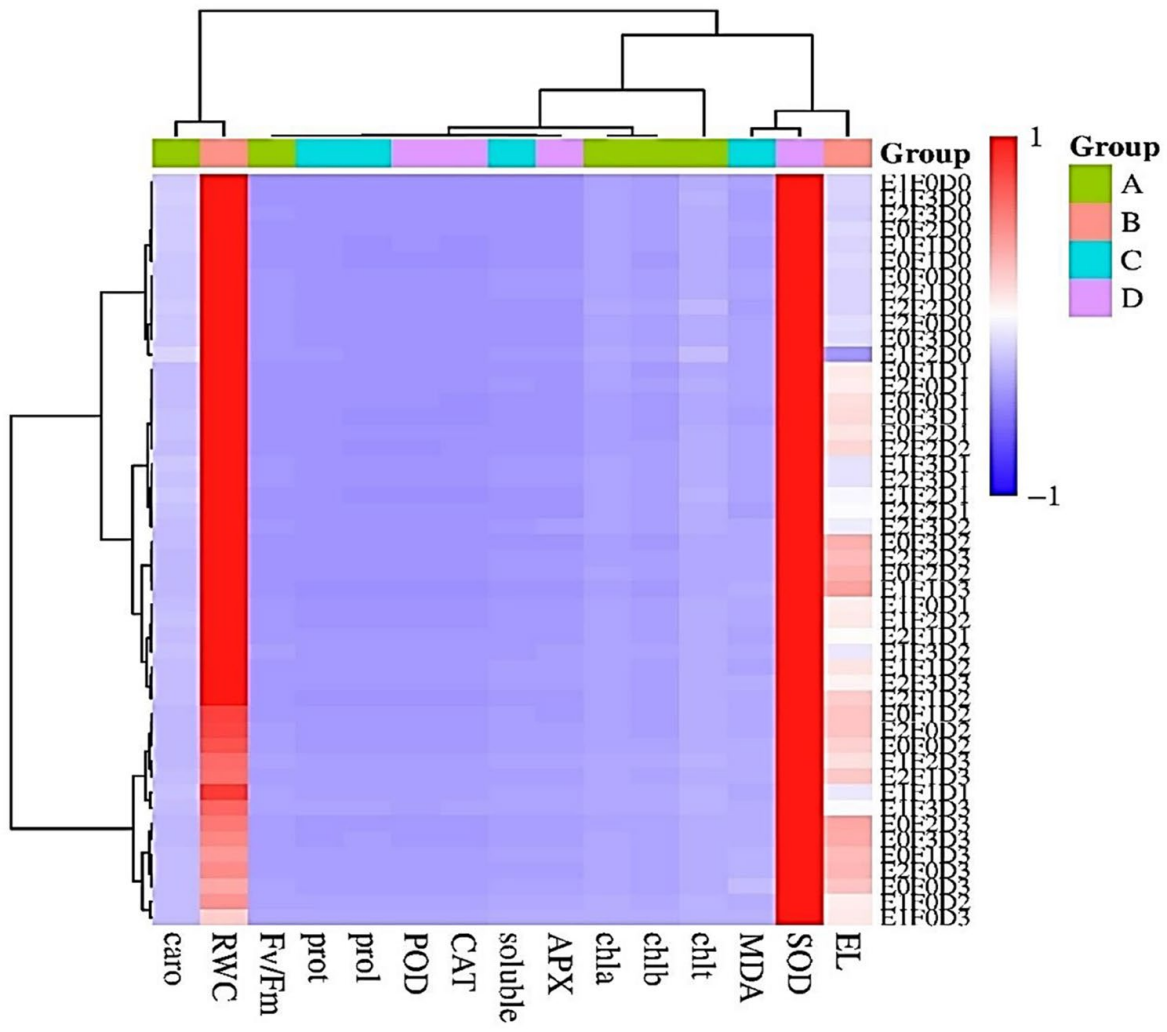
Using a heatmap of the Pearson correlation coefficients (r values) for the variable characteristics, hierarchical clustering analysis (HCA) was used to examine the correlations between the treatments, drought levels, and variable trait relationships in *T. vulgaris*. The r coefficient values (*r* = − 1 to + 1) are displayed on a colored scale, indicating positive (red) and negative (blue) correlations, respectively. *T. vulgaris* inoculated with various endophytic bacteria (E1), endophytic fungal (E2), and iron nanoparticles (F), under varying drought stress (D0-D3) conditions (100, 75, 50, and 25% FC). *Fv/Fm* maximum quantum efficiency of Photosystem II, *Chl a* chlorophyll a, *Chl b* chlorophyll b, and *Chl t* total chlorophyll, *caro* carotenoids, *El* electrolyte leakage, *RWC* relative water content, *prot* total protein, *prol* prolin, *soluble* soluble suga, *MDA* malondialdehyde, *CAT* catalase, *POD* peroxidase, *APX* ascorbate peroxidase, and *SOD* superoxide dismutas

Clustered heatmap illustrates various treatment groups, including endophyte at three levels (control (E0), EB (E1), and EF (E2), Fe NPs concentration at four levels (F0 - F3), and drought at three levels (D0 - D3). The colors and clustering lines indicated the proximity of similar treatments. It also showed different measured traits, including Fv/Fm, chl a, chl b, chl t, carotenoids, El, RWC, total protein, prolin, soluble sugar, MDA, CAT, POD, APX, and SOD. Numerical scale ranged from − 1 (dark blue) to + 1 (dark red), determining the extent of each trait, where red indicates a high value and blue indicates a low value. This heatmap revealed how each trait changed under different treatments. For example, SOD trait was very high in E1F0D3 treatment (dark red), but was lower in other treatments. Additionally, treatments with similar responses such as E2F3D0 and E0F2D0 were shown closer together and were clustered closely on the map. This type of heatmap is very useful for understanding how plants respond to different conditions and helps researchers better grasp the patterns in plant responses (Fig. 9).

#### Pearson correlation

**Fig. 10:**
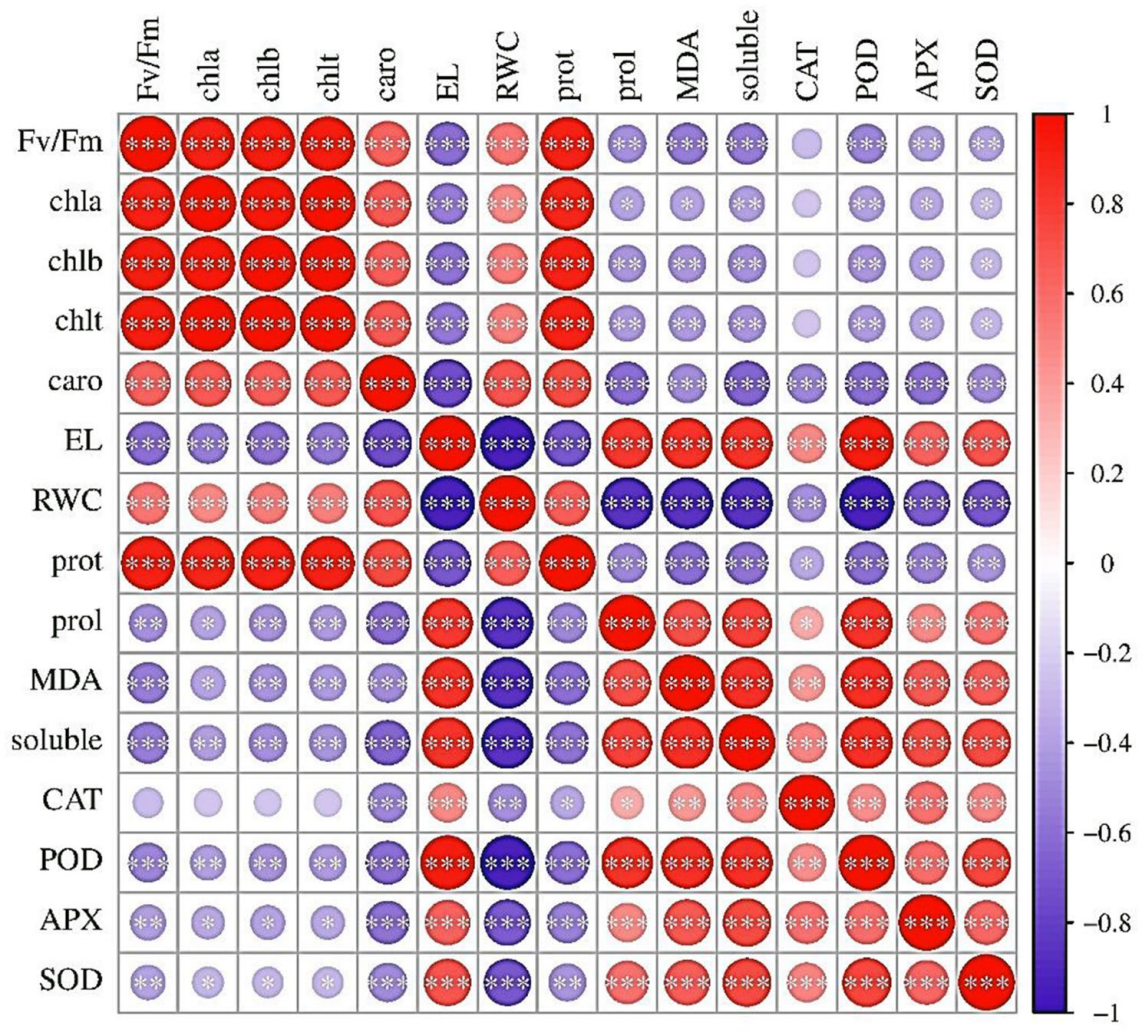
Using a Pearson correlation was used to examine the correlations between the treatments, drought levels and variable trait relationships in *T. vulgaris*. The r coefficient values (*r* = − 1 to + 1) are displayed on a colored scale, indicating positive (red) and negative (blue) correlations, respectively. Larger bullets represent higher values for the variable, whereas smaller bullets denote lower values. *T. vulgaris* inoculated with various endophytic bacteria (E1), endophytic fungal (E2), and iron nanoparticles (F), under varying drought stress (D0-D3) conditions (100, 75, 50, and 25% FC). *Fv/Fm* maximum quantum efficiency of Photosystem II, *Chl a* chlorophyll a, *Chl b* chlorophyll b, and *Chl t* total chlorophyll, *caro* carotenoids, *El* electrolyte leakage, *RWC* relative water content, *prot* total protein, *prol* prolin, *soluble* soluble sugar, *MDA* malondialdehyde, *CAT* catalase, *POD* peroxidase, APX ascorbate peroxidase, and *SOD* superoxide dismutas

The red color indicates a positive correlation and the blue color represents a negative correlation; the stronger the color intensity (dark red close to 1 and dark blue close to -1), the stronger the correlation. The strongest correlations were observed among chl a, chl b, and chl t. Additionally, strong correlations existed among CAT, POD, and SOD. Conversely, negative correlations were found among traits such as EL and RWC. As an indicator of oxidative damage, MDA had a negative correlation with RWC, indicating that an increase in MDA was associated with a decrease in RWC and increased membrane damage. These observations pointed to effects beyond the influence of endophytes and drought stress. Furthermore, Fe NPs (F0 - F3) and drought stress (D0 - D3) had significant impacts on most of the measured traits, as could be seen from diverse color patterns in the matrix (Fig. 10).

#### Principle component analysis (PCA)

A biplot is a two-dimensional plot used to display multivariate data. In this biplot (a), F1 and F2 axes explained 41.20% and 16.13% of data variance, respectively, together accounting for 57.33% of the total variance. In the second plot (b), the horizontal axis (F1) explained 57.34% and the vertical axis (F2) explained 16.43% of the total data variance. Together, these two axes covered 73.77% of the variance. This significant proportion indicated that these two principal components effectively captured data variations. F1 axis explained the highest variance and had the greatest impact on separating different combinations, while F2 axis covered the variance not explained by F1. The blue points represented different combinations of endophytes (E0, - E2), Fe NPs (F0 - F3), and drought levels (D0 - D3), with points closer together indicating combinations with similar effects on the measured traits. The red lines represented the direction and magnitude of the impacts of the measured traits, with the length of the lines indicating the effect strength of each trait on the combinations. The direction of the lines showed which combinations had the greatest effects on each trait.

**Fig. 11:**
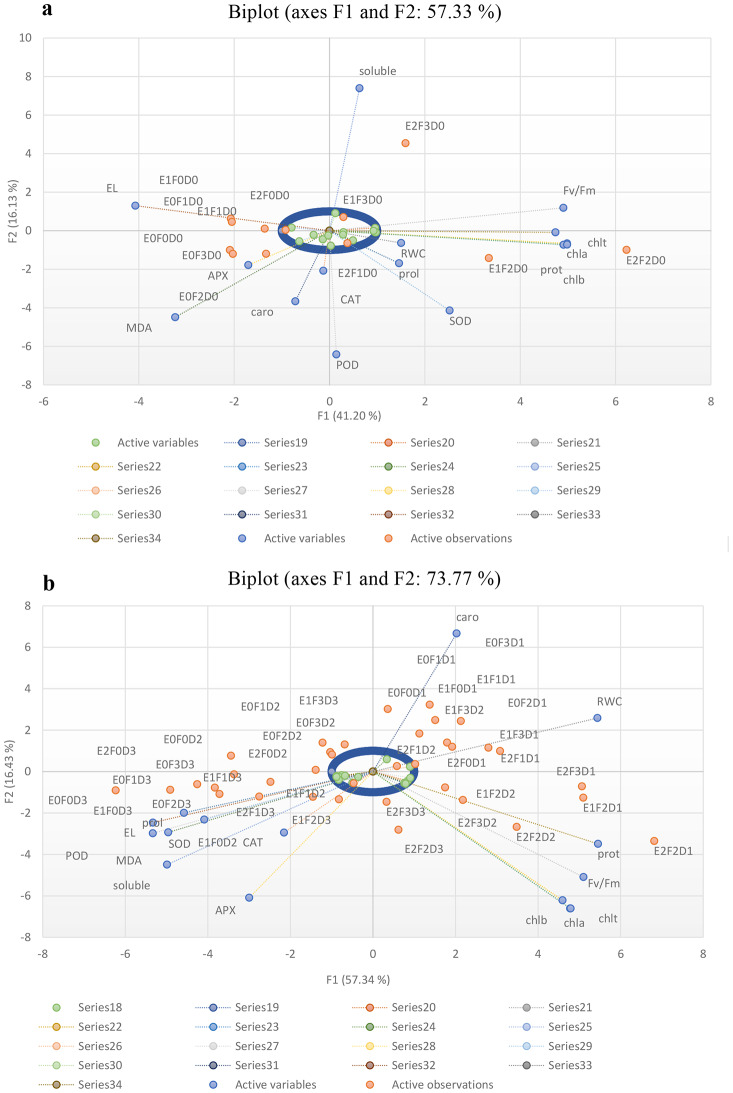
PCA-Biplots illustrating the relationship between treatments and various attributes of *T. vulgaris* under control (**a**) and drought stress (**b**) conditions (100, 75, 50, and 25% FC). (**a**) Biplot (axes F1 and F2: 57.33% of total variance); (**b**) Biplot (axes F1 and F2: 73.77% of total variance). *T. vulgaris* inoculated with various endophytic bacteria (E1), endophytic fungal (E2), and iron nanoparticles (F), under varying drought stress (D0-D3) conditions (100, 75, 50, and 25% FC). *Fv/Fm* maximum quantum efficiency of Photosystem II, *Chl a* chlorophyll a, *Chl b* chlorophyll b, and *Chl t* total chlorophyll, *caro* carotenoids, *El* electrolyte leakage, *RWC* relative water content, *prot* total protein, *prol* prolin, *soluble* soluble sugar, *MDA* malondialdehyde, *CAT* catalase, *POD* peroxidase, APX ascorbate peroxidase, and *SOD* superoxide dismutas

Features such as solubility and SOD had significant impacts on the distribution of data and their lines pointed to the right side of the plot, indicating that combinations in this area had higher levels of these features. Variables such as “MDA” and “EL” were located on the left side of the plot, indicating a different pattern of influence compared to other variables. The combinations E2F2D0 and E2F3D0 were clustered on the right side of the plot, likely exhibiting high levels of related features such as chl b or Fv/Fm ratio (Fig. 11a). Traits such as “RWC” and “caro” had stronger influences on the right side of the plot, indicating that combinations with higher values of these traits were located in this region. Variables such as “solubility,” “MDA,” and “EL” were positioned on the left side of the plot, suggesting a different pattern of influence, potentially indicating lower values of these traits. Combinations such as E2F2D1 and E2F3D2 were clustered more towards the bottom-right region of the plot, likely indicating higher levels of characteristics such as chl a, chl b, or Fv/Fm ratio. Generally, variables that were closer to the center of the plot had weaker impacts on the distribution of data (Fig. 11b).

## Discussion

Given the current context of climate change and global increase in demand for agricultural productivity, there is an urgent necessity to amplify the search for climate-smart remediation strategies that can mitigate water scarcity. Numerous studies have investigated the impacts of endophytes and NPs on abiotic stress resilience in plants, predominantly concentrating on enhancing plant performance.

Our study revealed that drought stress significantly impaired photosynthetic efficiency in *T. vulgaris*, as evidenced by the decreased Fv/Fm ratios observed at 50% and 25% FC. These findings complied with previous research indicating that drought stress adversely affected the photosynthetic apparatus by inducing stomatal closure, reducing CO_2_ availability, and disrupting chl synthesis [27]. Notably, applying Fe NPs, EB, and EF substantially improved Fv/Fm ratios under severe stress conditions. This enhancement in photosynthetic efficiency can be attributed to the role of Fe NPs in optimizing iron nutrition, which is crucial for synthesizing chl and maintaining photosynthetic machinery [10].

The capacity of CO_2_ absorption by plants is influenced by various environmental factors and drought reduces CO_2_ absorption rate [28]. In the present study, the percentage of foliar water found in the control and EF groups under drought stress was correlated with low photosynthetic efficiency and reduced CO_2_ absorption (Table 2). According to [29], when Robinia pseudoacacia plants were subjected to stress, those inoculated with *Rizophagus irregularis* remained stable in terms of CO_2_ absorption. Stress due to salt and drought can have similar effects, resulting in less water entering the plant [30]. The results obtained for *T. vulgaris* inoculated with endophytes were consistent with the data available in the literature. A study investigating the effects of inoculation with arbuscular mycorrhizal fungi (AMF) and EF on *Araucaria araucana* seedlings showed that these inoculations significantly increased survival rate under severe drought conditions (25% field capacity). Additionally, the maximum quantum efficiency of PSII (Fv/Fm) in the seedlings inoculated with AMF and the combination of EF + AMF were maintained at 0.71 and 0.64, respectively. This improvement in Fv/Fm was likely related to the optimization of Fe nutrition and preservation of photosynthetic integrity in the seedlings [31].

External application of NPs increased the contents of chl and carotenoids, as it significantly improved the structure of chl, light capture, pigment production, and rubisco activity, increasing the rate of photosynthesis in the plant [32].

In a study, the effect of Fe NPs on the photosynthetic performance of soybean under drought stress was investigated. The results showed that Fe NPs, particularly in combined nutritional method (seed priming + spraying), significantly improved the quantum efficiency of the photosynthetic system (FV/Fm). The use of Fe NPs was recognized as an effective nutritional technique for enhancing photosynthetic performance under drought stress in soybean [33].

The content of photosynthetic pigments, which are essential for photosynthesis, was better preserved in treated plants. The highest levels of chl and carotenoids were recorded in plants treated with EB and 1 mg L^− 1^ Fe NPs at 100% field capacity. This finding corroborated other studies indicating that Fe NPs were effective in maintaining the levels of various photosynthetic pigments under drought conditions [34].

Numerous investigations have substantiated the positive influences of employing EB and EF in sustaining and augmenting chl levels in plants subjected to drought stress conditions [35]. The protective role of endophytes likely involves the production of growth-promoting hormones and enhanced nutrient availability, such as N, P, and Fe, thereby mitigating chl degradation due to ROS [36]. This means that the combined application of Fe NPs and endophytes provides a synergistic effect, improving both nutrient availability and hormonal regulation, leading to enhanced photosynthetic performance under drought stress. In studies on the inoculation of *Araucaria araucana* seedlings with EF, a significant impact on chl t content was observed under drought stress (25% irrigation), resulting in maximum pigment concentrations [31]. Similar results were found in a study conducted on *Verbascum lychnitis* plants inoculated with a combination of endophytes, where the increase of photosynthetic pigments depended on the type of the used endophyte [37]. In a study, inoculation of *Nicotiana tabacum* with bacteria resulted in significant increases in chl a, chl b, chl t, and carotenoids in plants under drought stress. This increase in chl and carotenoid levels contributed to the enhancement of plant’s photosynthetic performance and indicated the positive role of endophytes in improving drought tolerance and maintaining the physiological quality of the plant [38].

Studies have shown that Se NPs could have a positive effect on increasing photosynthetic pigments in plants. Drought stress typically reduces chl and carotenoid pigments and negatively impacts photosynthetic processes. However, the application of Se NPs under drought conditions helps preserve and enhance pigment content by stimulating the activities of enzymes such as rubisco and stabilizing chloroplasts [39]. Fe NPs facilitate the absorption of essential metals such as Fe and Zn, which contribute to chl biosynthesis, thereby increasing the production of photosynthetic pigments. This improvement in pigment content, in turn, leads to increased photosynthetic efficiency and plant growth [39]. Studies have shown that NPs, particularly copper NPs (Cu NPs) and Se NPs, had positive impacts on the enhancement of photosynthetic pigments in plants. Under drought stress conditions, these NPs improved the quality and performance of strawberries. The mechanism of this effect included the increase of the content of chl pigments (chl a and chl b) and carotenoids, which helped improve photosynthetic processes. NPs stimulated enzymatic activities such as CAT and POD, increasing antioxidant activities and preventing the degradation of pigments. This improvement in pigments led to enhanced photosynthetic efficiency and plant growth [40]. Overall, NPs aided in the enhancement of photosynthetic pigment production by increasing the absorption of essential metals and supporting the stabilization of chloroplasts, thereby enhancing the ability of plants to cope with environmental stresses.

Electrolyte leakage, an indicator of cell membrane stability, was increased under drought stress but was significantly reduced with the application of Fe NPs and endophytes. This reduction was likely due to the improved water retention and antioxidative properties provided by these treatments, which protectetd membrane integrity [41].

Endophytes may produce exopolysaccharides and other osmoprotective substances that help in maintaining cell turgor and membrane stability under stress conditions [42]. Moreover, MDA content, an indicator of lipid peroxidation and cellular damage, was lower in treated plants, indicating reduced oxidative damage. The reduction of MDA levels corroborated the enhanced antioxidative defense provided by Fe NPs and endophytes [43]. In a study, endophytes, particularly *Aspergillus terreus*, presented significant positive impacts on reducing electrolyte leakage in plants under salt and drought stresses. These endophytes reduced oxidative activity by stimulating the production of antioxidant compounds and decreasing MDA contents in plants [44].

In a study, drought stress increased the levels of MDA, ion leakage, catalase, peroxidase, proline, and polyphenol oxidase in both mild and severe stress regimes. However, with the application of Fe NPs, pseudomonas and mycorrhiza, all measured traits showed the best performance under drought conditions. Additionally, they modulated the effects of drought stress and reduced its negative impacts [45].

In the present study, drought stress reduced RWC. However, the use of endophytes and Fe NPs could mitigate this reduction and improve water content in the plant. The mechanism of this effect was related to the increased production of compatible osmolytes, such as soluble sugars and proline, which helped maintain water balance and prevent severe water loss in plant tissues. These compounds enhanced the plant’s water retention capacity and reduced the negative effects of drought [46]. In a study, inoculation of endophytes (*Paenibacillus polymyxa* and *Fusarium oxysporum*) exhibited positive effects on relative water content in *Endostemon obtusifolius* under drought stress [46]. In a study, Si NPs had a positive effect on RWC in *Eleucine coracana* plant under drought stress. The use of Si NPs at concentrations of 50 and 100 mg L^− 1^ improved RWC. The mechanism of this effect was associated with enhanced activity of antioxidant enzymes such as SOD and APX, which helped maintain water balance and reduce oxidative stress in plant tissues. Additionally, Si NPs reduced MDA content and improved water retention capacity of the plant [47].

Protein content, often reduced under drought stress, was significantly higher in plants treated with Fe NPs and endophytes. This increase could be linked to improved nutrient uptake and metabolic activity facilitated by these treatments. The preservation of proteins, including enzymes, is critical for maintaining cellular functions under stress conditions [48].

Endophytes contribute to protein preservation by producing a range of bioactive compounds, including phytohormones such as cytokinins and gibberellins, which promote protein synthesis and inhibit protein degradation pathways. Additionally, endophytes can produce amino acids and peptides that directly supplement the plant’s protein pool. A noteworthy mechanism is the upregulation of target of rapamycin (TOR) pathway, which is a central regulator of cell growth and protein synthesis. Endophytes have been shown to activate TOR pathway, thereby enhancing protein biosynthesis and cellular growth under stress conditions [49]. In a study, EBs such as *Bacillus safensis* Ni7, were able to assist plants in coping with drought stress (equivalent to water potentials of -3 and − 6 bar) through improved nutrient uptake, increased protein production, and implementation of protective mechanisms. This bacterium possesses stress-responsive and protein production-related genes, such as catalase and superoxide dismutase, which were effective in reducing damage caused by drought stress [50].

Given the importance of iron as an essential micronutrient that acts as a cofactor for various enzymes involved in photosynthesis, respiration, and DNA synthesis, Fe NPs can enhance the synthesis and activity of these essential proteins by increasing the bioavailability of Fe. One of these proteins, ferritin, acts as an Fe-storage protein and can store and release Fe in a controlled manner, thereby helping to regulate Fe homeostasis and reduce oxidative damage. Additionally, studies have shown that nanohematite, after conversion from ferrihydrite to hematite, better addressed Fe deficiency in plants. This is due to changes in the structure and particle size of the NPs, which impact the availability of Fe to plants. Due to its smaller particle size and more regular crystalline structure, nanohematite can be more easily absorbed by the roots, thereby having a greater impact on improving Fe status in plants compared to nano-ferrihydrite. These structural and particle size changes make the use of Fe NPs as a suitable alternative to traditional Fe fertilizers an encouraging option for improving plant health and performance [51].

In a study conducted on quinoa (*Chenopodium quinoa*) under drought stress conditions, the effects of different nano-fertilizers on the protein content and physiological traits of the plant were investigated. In this research, drought levels included control (S1), mild drought stress (S2), and severe drought stress (S3), which were selected to evaluate the effects of different drought stresses on quinoa. Also, three levels of nano-fertilizers were used in this study, including control (F1), Si chelated nano-fertilizer (F2), and complete micro-chelated nano-fertilizer (2 gL^− 1^, F3). The results showed that treatment with Si chelated nano-fertilizer under severe drought stress (S3) resulted in the highest protein and proline contents, indicating good tolerance of the plant to drought stress. These positive effects wer mainly attributed to improved nutrient uptake and increased metabolic activities in the plant, which helped preserve and enhance essential proteins in the plant [52].

In a study conducted on Mungbean (*Vigna radiata*) under drought stress (50–60%, 70–80%, and 90–100% of field capacity), a significant increase was observed in proline accumulation (as an osmoprotectant and ROS scavenger) in plants subjected to severe drought stress [53]. Our results showed a significant increase in proline accumulation in plants treated with Fe NPs and endophytes under severe drought stress.

Endophytes can influence the variations of proline levels in Macadamia (*Macadamia tetraphylla* L.) under drought stress (7% soil water content (SWC) for 14 days). Increase of proline indicates the role of endophyte in osmotic regulation and improving the water status of the plant. However, the effect of endophytes varied depending on the type of the used strain, suggesting that the compatibility of plant and endophyte might determine their impact on proline accumulation [54]. Endophytes can stimulate proline synthesis in plants. In a study, plants inoculated with a combination of endophytes showed a high concentration of proline under drought stress (The plants were deprived of irrigation for two weeks), which helped improve drought tolerance [55].

NPs improve drought-induced reduction in carbon assimilation by increasing photosynthetic activity. Enhanced root growth, upregulation of aquaporins, modification of intracellular water metabolism, accumulation of compatible solutes such as proline, and ion homeostasis are the main mechanisms used by NPs to mitigate osmotic stress due to water deficit [56]. In a study, zinc oxide NPs led to a significant accumulation of proline in tomato (*Solanum lycopersicum*) plants, which contributed to the improvement of the nutritional content and health of the fruits [57]. In a study, Zn-chitosan-salicylic acid (ZCS) NPs had varying effects on proline levels in wheat plants under drought stress. At the concentration of 100 mg L^− 1^, proline levels were decreased. However, at concentrations of 200 and 400 mg L^− 1^, proline accumulation was increased, although plant growth was negatively affected. These results indicated the need to maintain an optimal level of proline for drought tolerance [58].

In this study, soluble sugar content was increased under drought stress and was also observed to increase in treated plants. Soluble sugars act as osmoprotectants, but their excessive accumulation can indicate severe stress. However, increase of soluble sugars in treated plants, due to the protective effects of Fe NPs and endophytes, might indicate a more stable physiological state and reduced negative impacts of stress. Endophytes might enhance the efficiency of carbohydrate metabolism, leading to better growth and reduced stress symptoms [59]. Similarly, the application of *Neocamarosporium phragmitis*, *Alternaria chlamydospora*, and *Microascus alveolaris* to *Lycium ruthenicum Murr* plants under drought conditions stress (30%, and 60% of field capacity) increased glutathione content and SOD activity as well as soluble, protein and proline contents [60]. In a review study, the effects of dark septate sndophytes (DSEs) on mitigating the damage caused by ROS in plants under abiotic stresses were examined. The findings revealed that DSEs enhanced the activity of antioxidant enzymes such as SOD. Additionally, these endophytes boosted the production of compounds such as polysaccharides, soluble sugars, proline, and glutathione, which act as compatible osmolytes. These compounds contributed to maintaining water balance in plants and protected plant cells under the conditions of drought, salinity, and reduced nutrient availability in the soil. Consequently, this process improved plant tolerance and increased the levels of soluble sugars in the plants [59].

In a review article, the use of NPs, particularly TiO_2_, significantly enhanced the accumulation of proline, glycine betaine, soluble sugars, and total proteins, thereby improving plant growth under drought stress [61]. In the present study, the interactions of Fe NPs and endophytes synergistically enhanced the plant’s antioxidant defense mechanisms and reduced proteolysis typically caused by oxidative stress. This was achieved by increasing the expression of antioxidant enzymes such as SOD, CAT, POD, and APX, which reduced the damage caused by ROS and stabilized cellular proteins [62, 63]. Endophytes help the accumulation of these compounds in plant tissues by regulating the expression of genes related to antioxidant biosynthesis (such as MIOX, crtB, gpx), while simultaneously reducing the expression of genes associated with antioxidant metabolism (such as GST, PRODH, ALDH). These processes improved defense system in plants against oxidative stress [62]. Epichloë endophytes augmented the levels of APX, CAT, and GR in *Festuca arizonica* and *Achnatherum robustum* plants subjected to drought stress (30%, and 60% of field capacity) [62].

The occurrence of Epichloë in drought-treated *E. dahuricus* plants elevated the levels of APX, CAT, POD, and SOD antioxidants [64]. Furthermore, the antioxidants CAT, POD, and polyphenol oxidase (PPO) were more elevated in endophyte-infected perennial ryegrass (*Lolium perenne*) plants in comparison to endophyte-free plants cultivated under water deficit conditions (30% SWC) [64]. Consistent with our hypothesis, the elevated antioxidant levels in *T. vulgaris*-mediated plants at low soil moisture were associated with diminished oxidative damage [65].

NPs can have positive effects on the antioxidant activities of plants under drought and salinity stresses. These NPs enhance the plant’s defense mechanisms by protecting cell membranes and improving photosynthetic apparatus. They optimize antioxidant activity by increasing nutrient uptake and regulating the levels of hormones and phenolic compounds. This enhancement in antioxidant activity helps reduce the damage caused by oxidative stress and increases the resilience of plants to stressful conditions [5].

Fe NPs have been investigated as an innovative technique in agriculture to alleviate stress caused by Fe deficiency in plants, particularly in grapevines. A study demonstrated that the application of Fe NPs at various concentrations (up to 10 micromoles) enhanced the activity of antioxidant enzymes such as CAT, glutathione peroxidase (GPX), and glutathione reductase (GR). Furthermore, Fe NPs contributed to the protection of plants against oxidative stress by improving chl content and fluorescence in drought-stressed plants. These results indicated the significant potential of Fe NPs in enhancing plant performance and mitigating the negative effects of environmental stresses [66].

In this study, the results showed that for all traits except for EL, MDA, and soluble sugar, EB had greater effects on improving the traits and performance of plants compared to EF, which was consistent with other studies. Additionally, for all traits (except for CAT, APX, electrolyte leakage, and soluble sugar), the use of Fe NPs at concentration of 1 mg L^− 1^ led to significant improvements in these traits. However, at concentrations of higher than 1.5 mg L^− 1^, a decrease in the measured traits was observed due to toxicity. These findings indicated that selecting appropriate concentrations of Fe NPs could enhance the physiological and biochemical characteristics of plants, while higher concentrations might have negative effects.

The biplot analysis used in this study successfully elucidated the complicated interaction between endophytes and FeNPs in their impact on plant growth in plants. The results reveal a synergistic action of this combination, with enhanced physiology in plants and stress tolerance. The strains used in this study showed great ability to adapt to adverse conditions and are very appropriate for sustainable agriculture [67]. Deposition of their genetic information in international repositories has made these findings more usable on a larger scale. The study opens doors towards innovative agricultural practices and sets the stage for future research in the area.

## Conclusion

The present study assessed a combined approach based on application of nanoparticles and symbiotic microorganisms to *T. vulgaris*. The results revealed that these treatments not only supported activity of antioxidant enzymes but also promoted accumulation of essential compounds, including protein, soluble sugars, and proline, all of which contributed to drought stress tolerance in the plant. The study showed that sustaining higher levels of chl and carotenoids, as well as higher relative water content, greatly contributed to the well-being of this plant when water was limited. The study also cautioned against using too high concentrations of NPs, as these may cause phytotoxicity and counteract the effects. The approach may prove to be an excellent asset in sustainable agriculture, especially where there are concerns about increasing water scarcity and climate variability. Future research should aim to enhance these applications to provide maximum yield with minimum risks.

## Methods

### Plant sampling and sterilization

This study focused on identifying the natural habitats of species belonging to the Lamiaceae family in Hormozgan Province, Iran. For each plant species, three separate specimens were gathered at every sampling location. The collected plant materials were securely stored in sterilized polyethylene bags and transported to the lab in an insulated cooler kept at a constant 4 °C with ice packs. Taxonomic verification of the plant samples was conducted by Dr. Mohammad Amin Soltanipour at Agricultural and Natural Resources Research Center of Hormozgan province herbarium facility, which also serves as the repository for these preserved plant specimens. Samples of Lamiaceae plants, along with their deposition number (Table 3) were collected from various locations in Hormozgan Province, Iran, at geographical coordinates 27.4173° N, and 56.1422° E.

**Table 3:** List of collected plant voucher specimens with corresponding herbarium accession codes

No.	Voucher specimen	Deposition number
1	*Menta mozaffarianii*	6331
2	*Teucrium pollium*	6307
3	*Zataria multiflora*	6297
4	*Lavandula stricta*	6312
5	*Zhumeria majdae*	6320
6	*Otostegia persica*	6323
7	*Salvia mirzayanii*	6354
8	*Teucrium stockisanum*	4064
9	*Teucrium orientale*	6295

For surface sterilization of leaves, stems and roots, a modified protocol [68] was utilized. Additionally, the sterilization and isolation of endophytic microorganisms from Lamiaceae plants were performed [69].

### Isolation of bacterial endophytes

**Fig. 12:**
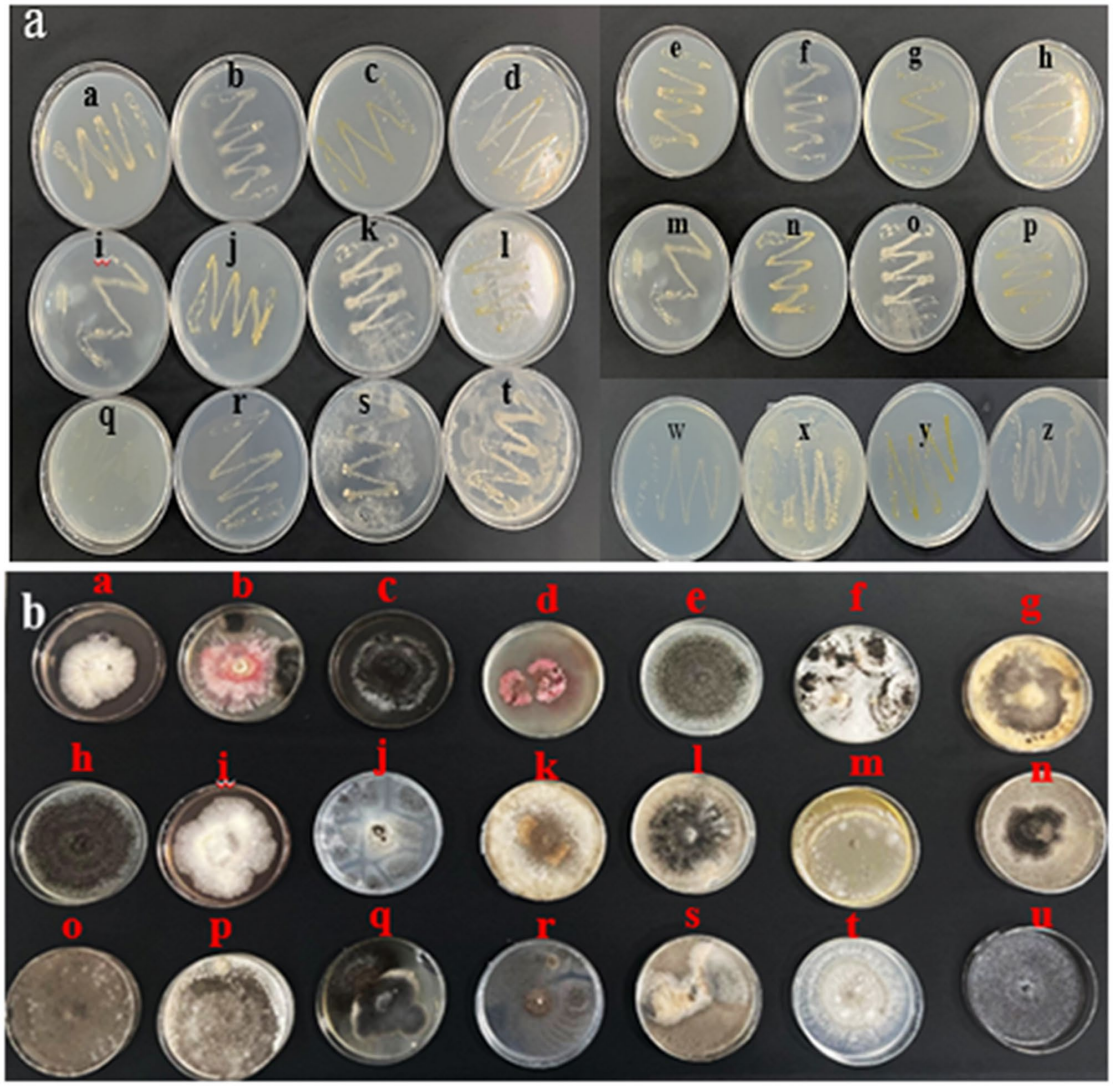
NA culture media for the growth of bacterial endophytes (**a**) PDA culture media for the growth of fungal endophytes (**b**)

To evaluate the sterilization efficiency, 0.1 mL of the final rinse water was cultured on NA (Nutrient Agar) medium and examined for microbial growth. Subsequently, 0.5 g of leaves from each species were crushed using a sterile mortar and pestle, converted into a suspension, and mixed with 30 mL of sterile distilled water. The samples were then placed on a shaker at 120 rpm and 25 °C for 1 h. Afterwards, 0.1 mL of the solution was plated in triplicate on NA media supplemented with nystatin (25 µg·mL^− 1^) to suppress fungal growth, and incubated in the dark at 35 ± 2 °C. The cultures were observed for bacterial growth, for 72 h. Following initial growth, bacterial isolates were streaked onto fresh NA agar plates to isolate pure colonies (Fig. 12a). Selected single colonies were then transferred to NB (nutrient broth) tubes and stored in 50% (v/v) glycerol at -80 °C [70].

### Molecular identification of bacterial endophyte

The identity of the studied bacterial isolates was determined using 16 S rRNA gene sequencing. In the initial phase, genomic DNA was extracted through an optimized protocol based on the standard method by Lutz et al. [71]. Target region amplification was performed using the universal primer pair 27 F (5′-AGAGTTTGATCCTGGCTCAG-3′) and 1492R (5′-GGTTACCTTGTTACGACTT-3′), which specifically target the conserved regions at the beginning and end of the 16 S rRNA gene, respectively. The polymerase chain reaction (PCR) was conducted under optimized conditions containing 1× reaction buffer, 1.2 mM magnesium chloride as an enzymatic cofactor, 3 units of thermostable DNA polymerase, 0.3 mM deoxynucleotide triphosphate mixture, and each primer at 0.6 µM concentration. Approximately 7.5 ng of bacterial genomic DNA was used in a final reaction volume of 25 µL. The thermal cycling protocol commenced with an initial denaturation step at 94 °C for 3 min. followed by 35 cycles of DNA amplification. Each amplification cycle consisted of three steps: denaturation at 96 °C for 1 min to separate the DNA strands, primer annealing at 55 °C for 1 min to allow primer binding, and extension at 72 °C for 1 min for DNA synthesis. A final extension step was carried out at 72 °C for 10 min to complete the amplification. Following quality and size verification of PCR products through 1% agarose gel electrophoresis, selected amplicons were subjected to sequencing. Post-sequencing bioinformatic analyses included sequence comparison against the GenBank database using BLAST tools.

### Isolation of fungal endophytes

Fungal cultivation was initiated by inoculating four replicate segments from each specimen onto potato dextrose agar (PDA) amended with chloramphenicol 150 mg l^− 1^. The Petri dishes were sealed with parafilm and maintained at 28 °C for incubation. As a sterilization control, parallel samples of non-sterilized tissues (rinsed solely with distilled water) were processed identically. Regular monitoring occurred at 72-hour intervals to assess fungal development. Following an incubation period of 3–4 weeks, endophytic fungi were purified through hyphal tip isolation, where advancing hyphal fronts from colony margins were aseptically transferred to new PDA plates to establish axenic cultures (Fig. 12b) [72].

### Molecular identification of fungal endophyte

Genomic DNA isolation was performed following standardized CTAB protocols [73]. For precise species discrimination, the ITS region of ribosomal DNA was amplified employing primers ITS1 (5′-TCCGTTGGTGAACCAGCGG-3′) and ITS4 (5′-TCCTCCGCTTATTGATATGC-3′). The PCR amplification protocol was initiated with a denaturation at 96 °C for 3 min, followed by 33 cycles consisting of denaturation at 95 °C for 30 s, annealing at 55 °C for 50 s, and extension at 72 °C for 2 min, culminating in a final extension at 72 °C for 10 min. The resulting amplicons were sequenced via the Sanger method, and the obtained sequences were analyzed using Geneious Prime software. Fungal identification was performed by comparing the sequences to the NCBI database using BLAST.

### Creating microbial solutions for thyme treatment

For fungal inoculation, a specific fungal strain was grown on PDA in a controlled environment set at 26 ± 1 °C with equal 12-hour light/dark cycles for three weeks. Once mature, the fungal spores were carefully harvested by rinsing the culture plates with a sterile solution containing 20% Tween-20 in distilled water. The spore concentration was then measured using a hemocytometer and adjusted to 1 × 10⁶ CFU/mL for consistent application [74].

For bacterial inoculation, a selected bacterial strain was cultured in nutrient broth (NB) under 28 ± 1 °C with continuous shaking at 120 rpm, maintaining the same 12-hour light/dark cycle. After 24 h, the bacterial density was calibrated spectrophotometrically to reach 1 × 10⁸ CFU/mL, ensuring optimal viability for plant treatment. Subsequently, the drought tolerance of both fungal and bacterial isolates was evaluated under controlled laboratory conditions [75].

### Plant material

In this research, two drought-resistant microorganisms were utilized. The bacterium *Azospirillum lipoferum* (accession number PP837752) was isolated from the root system of Salvia mirzayanii, while the fungus *Aspergillus oryzae* (accession number PP840055) was obtained from the aerial parts of the same plant. Preliminary evaluations revealed that these strains exhibited the highest tolerance to water-deficient conditions compared to other isolates and were therefore selected for further experimentation.

### Treating thyme seeds using endophytes

The thyme seeds were obtained from Hatam Agricultural Growth Company in Shiraz, Iran. To sterilize them, the seeds were first rinsed with 70% ethanol for 30 s and then treated with 0.5% sodium hypochlorite for 1.5 min. Afterward, they were rinsed three times with sterile water. This entire sterilization process was repeated twice to ensure complete sterilization and uniformity among all samples. All sterilization and handling procedures were performed under a laminar flow hood for aseptic conditions [76]. To begin the experimental procedure, 1% carboxymethyl cellulose solution was created to improve seed-endophyte association. Sterilized seeds were soaked in the endophyte suspension and agitated for four hours, while seeds soaked in sterile distilled water functioned as the control. Following inoculation, seeds were planted in propagation trays containing a sterilized (121 °C, 15 min) peat moss-perlite substrate. The trays were incubated in Hormozgan University’s greenhouse (27°16’09.8"N 56°18.6"E) under regulated environmental parameters maintaining 20–25 °C and 65–70% relative humidity, with photosynthetically active radiation (PAR) ranging from 700 to 950 µmol m⁻² s⁻¹ during the 12-hour daylight period. The greenhouse maintained a strict 12-h light/12-h dark photoperiod.

### Treating thyme plants using endophytes

Following an initial two-month growth period during which seedlings exhibited satisfactory development, they were transplanted into larger-volume containers. The endophytic treatment protocol was initiated by the fourth month of cultivation, when plants had typically developed four lateral branches. Microbial application (For each *T. vulgaris* plant, 10 mL of fungal endophyte suspension and 10 mL of bacterial endophyte suspension were separately sprayed onto the leaves) was performed in two distinct stages over one month, enabling systematic comparison of drought stress responses between endophyte-inoculated plants and control specimens.

### Genetic confirmation of endophyte establishment

Following the final inoculation, tissue samples (leaves, stems, and roots) were collected from six thyme plants to molecularly confirm endophyte establishment. After surface sterilization, samples were cultured on selective media: NA agar for bacterial endophytes and PDA for fungal endophytes. Following incubation, putative endophytic colonies were PCR-amplified using taxon-specific markers (16 S rRNA gene for bacteria [77] and ITS region for fungi [78]). The PCR products were sequenced and compared against the GenBank database using BLAST analysis. Control plants underwent identical isolation procedures to exclude external microbial contamination, with PCR product purity verified through universal primer amplification.

#### Preparation of fenps suspension

The FeNPs used in this study were alpha-phase iron oxide nanoparticles (Fe₂O₃, ≥ 98% purity, 20–40 nm particle size) obtained from Pishgaman Nano Mavad, a nanomaterials company based in Mashhad, Iran (CAS number: 1309-37-1). Preparation of the FeNPs suspension was carried out by dispersing FeNPs at concentrations of 0.5, 1, and 1.5 mg L⁻¹ in one liter of distilled water. The mixture was then agitated using a Thermo Scientific MaxQ 4000 shaker at 120 rpm for 10–15 min to ensure a homogeneous and uniform suspension. Once fully mixed, the suspension was transferred into appropriate containers for later use in spraying applications.

### Multi-Factorial study on irrigation, fenps, and endophytes

The experiment was evaluated as a factorial experiment in a completely randomized design with three replications. evaluating three treatment factors: four irrigation levels (100%, 75%, 50%, and 25% of field capacity), four FeNPs concentrations (0, 0.5, 1, and 1.5 mg L⁻¹), and three endophyte treatments (control, bacterial, and fungal inoculation). At 50% flowering stage, thyme plants were harvested and subsequently dried in shade for 5 days.

### Drought tolerance test

To perform screening test, various methods, including biochemical and genetic tests, were used. One of these tests was to examine the tolerance of isolates to dryness. After isolating, purifying, and identifying the bacteria, to assess the tolerance of the isolates to different levels of dryness, NB and PDA media containing concentrations of 0, 152, 224, and 280 g of polyethylene glycol (PEG) 6000 per liter of NB and PDA media were used. Water potentials of these concentrations were equivalent to 0, -3, -6, and − 9 bars, respectively. To determine water potential in terms of bars, different amounts of PEG in gr/L were calculated using the following equation [79]: (1)

where a, b, and c are empirically derived constants for PEG 6000 and the specific temperature.

Alternatively, a reference table may be used for direct values.

1 $${\rm{\Psi = a - b }}\left( {{\rm{PEG\:concentration}}} \right){\rm{ + c }}{\left( {{\rm{PEG\:concentration}}} \right)^{\rm{2}}}$$

### Photosynthetic parameters

chl a, chl b, and chl t contents were measured following the method proposed by Straumite et al. [80] with slight modifications. Acetone was utilized as solvent and a spectrophotometer was employed to read the absorptions of samples at wavelengths of 662 and 645 nm. The total carotenoid content in thyme plant extracts was quantified using HPLC-DAD method and was expressed as 100 mg g^− 1^ DW. Extraction was performed through sequential solvent extraction employing different techniques [81]. The measurement of maximum quantum yield of PSII (Fv/Fm) was conducted using a chlorophyll fluorometer (OS-30p model, manufactured in the United States). For this purpose, parameters including ground fluorescence (F0), maximum fluorescence (Fm), consequently variable fluorescence (Fv), and Fv/Fm were calculated.

### Determination of total protein, proline and soluble sugar contents

Rekowski et al. [82] assay was employed to measure protein concentration. Bovine serum albumin (BSA) standards were used for calibration and absorbance at 595 nm was measured. The resulting standard curve facilitated determination of protein contents in plant extracts. Proline contents in plant samples was assessed using the method outlined by Forlani and Funck [83]. Leaf tissues underwent homogenization and extraction with sulfosalicylic acid. Proline in the samples reacted with acid ninhydrin, producing a chromophore, and the color of the product was measured at 520 nm. Proline concentration was quantified using a standard curve generated with known proline concentrations. Soluble sugars in plant samples were determined according to Mo et al. [84]. Plant tissues were homogenized and the extraction of soluble sugars was carried out with ethanol. Anthrone reagent was introduced to the extract, resulting in the development of a blue-green color indicative of the presence of sugars. The absorbance of the solution was measured at 620 nm and the concentration of soluble sugars was quantified based on a standard curve derived from known sugar concentrations.

### Relative water content (RWC)

To determine RWC, fully developed leaves were promptly harvested and weighed to record fresh weight (FW). Subsequently, the leaves were immersed in distilled water for 4 h, and their turgid weight (TW) was calculated. Afterward, the leaves were subjected to drying in an oven at 65 °C for 24 h to obtain dry weight (DW). RWC was then calculated using the following equation [85]: (2)

2 $${\rm{RWC}}\>\left( {\rm{\% }} \right)\>{\rm{ = }}\>{{{\rm{FW - DW}}} \over {{\rm{TW - DW}}}}\>{\rm{ \times }}\>\>{\rm{100}}$$

### Lipid peroxidation

The degree of oxidative lipid degradation was assessed by quantifying MDA content and percentage of electrolyte leakage (EL) in leaves. MDA content in plant samples was assessed using the method devised by Leon and Borges [86]. Leaf tissues underwent homogenization and the resulting supernatant was combined with thiobarbituric acid (TBA) reagent. Following a heating process, the development of a pink color, indicative of the reaction of MDA and TBA, was measured at 532 nm. The content of MDA was determined using a standard curve with known MDA concentrations.

EL in plant samples was assessed following the procedure described by Shi et al. [87]. Fully developed leaves were collected, washed, and immersed in deionized water for 24 h to determine initial electrolyte leakage (ELi). Subsequently, the samples underwent autoclaving and final electrolyte leakage (ELf) was measured after an additional 24 h. The percentage of electrolyte leakage was then calculated using the following equation: (3)

3 $${\rm{EL}}\>\left( {\rm{\% }} \right){\rm{ = }}{{{\rm{ELi}}} \over {{\rm{ELf}}}}{\rm{ \times 100}}$$

### Antioxidant enzyme activities

To measure the activity of antioxidant enzymes in plants, enzyme extracts were prepared using the following procedure. Fully developed plant tissues were harvested and immediately homogenized in a cold extraction buffer containing Tris-HCl (pH 7.5), EDTA, and other necessary reagents. The homogenate was centrifuged at a low temperature to separate the soluble fraction. The resulting supernatant, containing enzyme extract, was then used for the quantification of antioxidant enzyme activities.

CAT activity was assessed following the method outlined by Jamshidian et al. [88]. The reaction mixture, consisting of 15 mM H_2_O_2_, 50 mM phosphate buffer (pH 7.0) and 50 µL enzyme extracts was used to measure CAT activity. The enzyme’s activity was determined by monitoring the reduction of absorbance at 240 nm due to the consumption of H_2_O_2_.

POD activity in plants was determined following the method outlined by Rai et al. [89]. Guaiacol and H_2_O_2_ were added to the plant extract and the reaction mixture was thoroughly mixed and incubated at a specific temperature for a designated period. Increase of absorbance at 470 nm was measured using a spectrophotometer, indicating the conversion of guaiacol to tetraguaiacol due to peroxidase activity.

APX enzyme activity was determined using the methodology described by Ranieri et al. [90]. The reaction involved the interactions of ascorbate peroxidase, ascorbic acid, and H_2_O_2_, resulting in the production of dehydroascorbate, which was quantified at a wavelength of 290 nm. The reaction mixture comprised 300 µL 0.1 mM EDTA, 750 µL 50 mM phosphate buffer, 200 µL 0.5 mM ascorbic acid, 200 µL 35% H_2_O_2_, and 25 µL enzyme extract.

The method developed by Amiri Bahmanbiglo and Eshghi [91] was employed to evaluate the activity of SOD in plants. The reaction mixture contained 50 mM phosphate buffer (pH 7.8), 13 µM methionine, 75 µM p-nitro blue tetrazolium chloride (NBT), 1.3 µM riboflavin, and 40 ml enzyme extract. This mixture underwent incubation under light conditions to induce the generation of superoxide radicals. One unit of SOD activity was defined as the enzyme quantity necessary to achieve 50% inhibition in the rate of NBT reduction measured at 560 nm.

### Statistical analysis

Data was analyzed using SAS software version 9.4 and significant differences were determined using LSD test. Parametric tests were used for data analysis. The mean values of three replications plus standard deviation (M ± SD) were reporeted as the results. The analysis, conducted using XLSTAT software and Shapiro–Wilk test, revealed a P value of ≥ 0.05, indicating that our data adhered to a normal distribution. Consequently, parametric tests were employed for data analysis [92].

### Principal component analysis (PCA)

Principal component analysis (PCA) was performed to visualize the relationships among bacterial isolates and various attributes of *T.vulgaris* under control and drought stress conditions. Cluster analysis and PCA were conducted using XLSTAT software, version 2020 (www.xlstat. com). PCA-biplot was drawn to display the distribution of samples and the loading of each attribute in the principal component space.

### Heatmap and correlation analyses

HCA was conducted using SRPLOT (online) [93].

## Supplementary Information

Below is the link to the electronic supplementary material.

Supplementary Material 1

Supplementary Material 2

## Data Availability

Sequence data that support the findings of this study have been deposited in the NCBI database with accession numbers PP837752 (Azospirillum lipoferum) and PP840055 (Aspergillus oryzae).

## Abbreviations

FeNPs: Fe_2_O_3_ nanoparticles
EB: Endophytic bacteria
EF: Endophytic fungi
CAT: Catalase
POD: Peroxidase
PPO: Polyphenol oxidase
APX: Ascorbate peroxidase
SOD: Superoxide dismutase
TOR: Target of rapamycin
PCR: Polymerase Chain Reaction
F0: Initial Fluorescence
Fm: Maximum Fluorescence
Fv: Variable Fluorescence
DS: Drought Stress
MDA: Malondialdehyde
RWC: Relative Water Content
PCA: Principal Component Analysis
EC: Electrical Conductivity
DW: Dry Weight
FW: Fresh Weight
TBA: Thiobarbituric Acid
EL: Electrolyte Leakage
NBT: Nitroblue Tetrazolium
HCA: Hierarchical Clustering Analysis
AsA: Ascorbic acid
SeNPs: Selenium nanoparticles
SWC: Soil water content
Mn: Manganese
Cr: Chromium
MIOX: Myo-Inositol Oxygenase
crtB: Carotenoid Biosynthesis
gpx: Glutathione Peroxidase
GST: Glutathione S-transferase
PRODH: Proline dehydrogenase
ALDH: Aldehyde dehydrogenase
