# Supplementary figures and images for: How thyme thrives under drought: insights into photosynthetic and membrane-protective mechanisms
Source: BMC Biotechnol. 2025 Sep 2;25:95. doi: 10.1186/s12896-025-01026-9 (PMC12403968; doi:10.1186/s12896-025-01026-9)

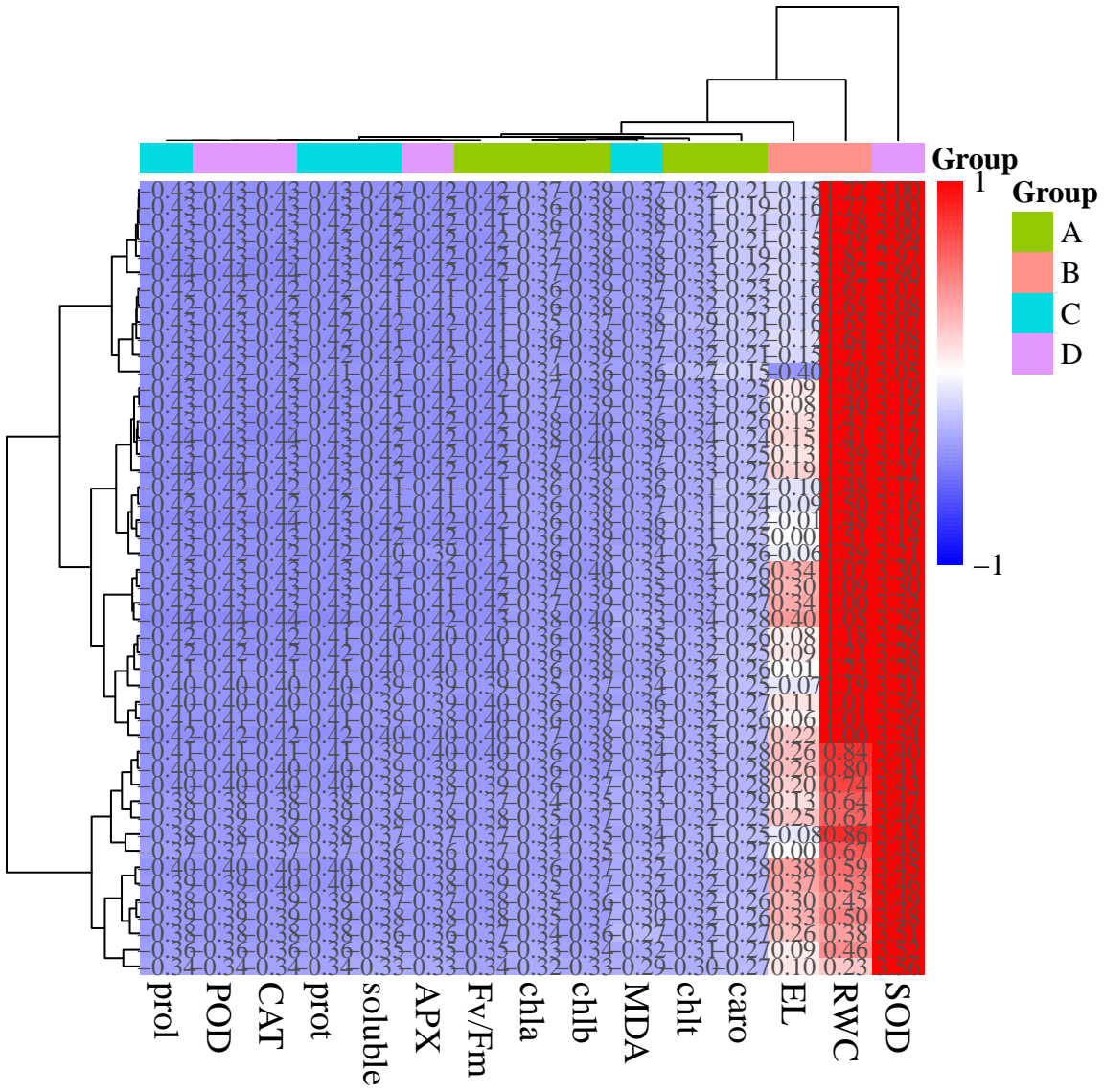

Supplement: Supplementary file 1 — Supplementary Material 1 [file 12896_2025_1026_MOESM1_ESM.pdf]

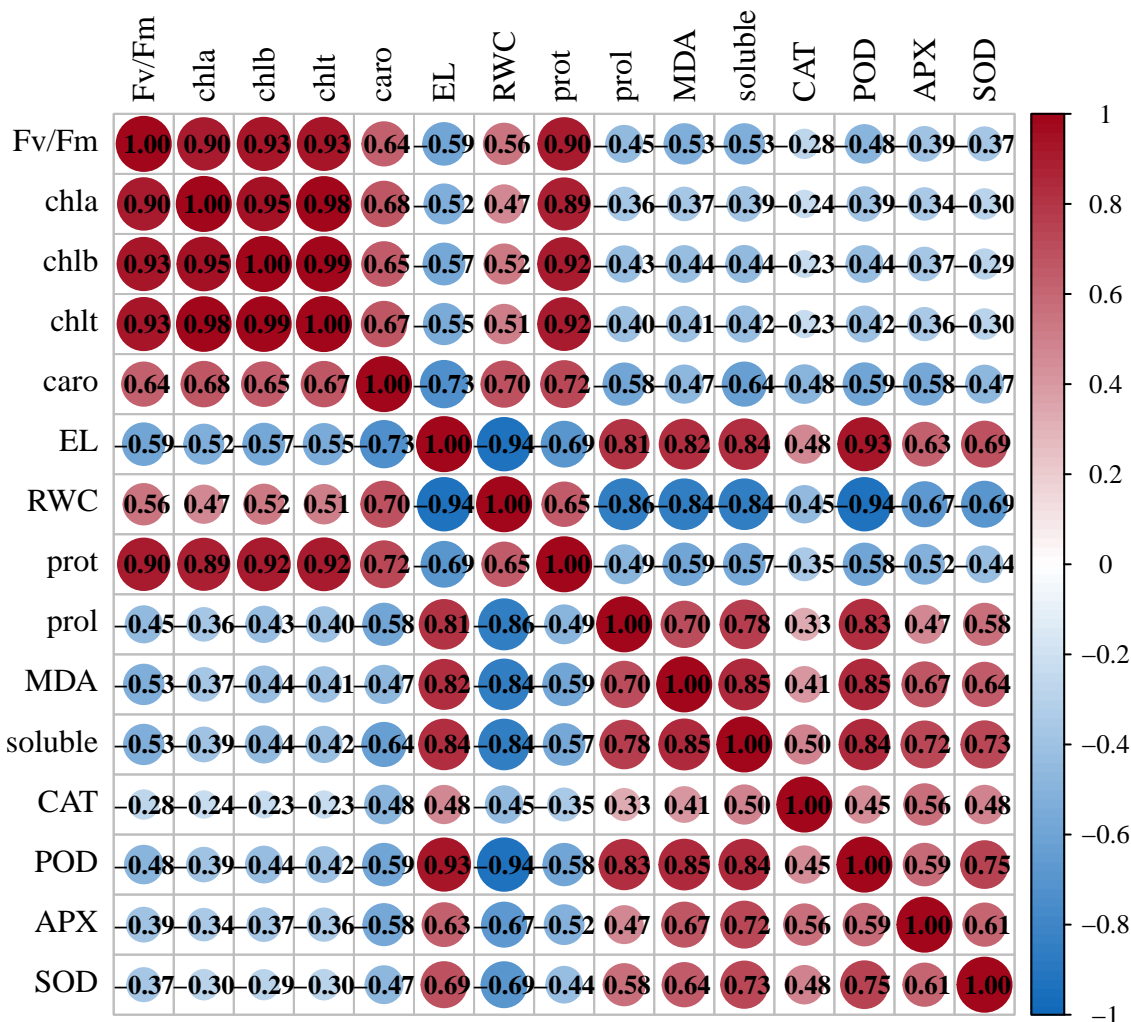

Supplement: Supplementary file 2 — Supplementary Material 2 [file 12896_2025_1026_MOESM2_ESM.pdf]
